# PTBP3 Mediates IL‐18 Exon Skipping to Promote Immune Escape in Gallbladder Cancer

**DOI:** 10.1002/advs.202406633

**Published:** 2024-08-08

**Authors:** Cheng Zhao, Jing‐wei Zhao, Yu‐han Zhang, Yi‐di Zhu, Zi‐yi Yang, Shi‐lei Liu, Qiu‐yi Tang, Yue Yang, Hua‐kai Wang, Yi‐jun Shu, Ping Dong, Xiang‐song Wu, Wei Gong

**Affiliations:** ^1^ Laboratory of General Surgery and Department of General Surgery Xinhua Hospital affiliated with Shanghai Jiao Tong University School of Medicine No. 1665 Kongjiang Road Shanghai 200092 China; ^2^ Shanghai Key Laboratory of Biliary Tract Disease Research No. 1665 Kongjiang Road Shanghai 200092 China

**Keywords:** alternative splicing, gallbladder cancer, IL‐18, immune escape, PTBP3

## Abstract

Gallbladder cancer (GBC) is the most common malignant tumor of the biliary system, with poor response to current treatments. Abnormal alternative splicing has been associated with the development of a variety of tumors. Combining the GEO database and GBC mRNA‐seq analysis, it is found high expression of the splicing factor polypyrimidine region‐ binding protein 3 (PTBP3) in GBC. Multi‐omics analysis revealed that PTBP3 promoted exon skipping of interleukin‐18 (IL‐18), resulting in the expression of ΔIL‐18, an isoform specifically expressed in tumors. That ΔIL‐18 promotes GBC immune escape by down‐regulating FBXO38 transcription levels in CD8+T cells to reduce PD‐1 ubiquitin‐mediated degradation is revealed. Using a HuPBMC mouse model, the role of PTBP3 and ΔIL‐18 in promoting GBC growth is confirmed, and showed that an antisense oligonucleotide that blocked ΔIL‐18 production displayed anti‐tumor activity. Furthermore, that the H3K36me3 promotes exon skipping of IL‐18 by recruiting PTBP3 via MRG15 is demonstrated, thereby coupling the processes of IL‐18 transcription and alternative splicing. Interestingly, it is also found that the H3K36 methyltransferase SETD2 binds to hnRNPL, thereby interfering with PTBP3 binding to IL‐18 pre‐mRNA. Overall, this study provides new insights into how aberrant alternative splicing mechanisms affect immune escape, and provides potential new perspectives for improving GBC immunotherapy.

## Graphical abstract

PTBP3 mediated IL‐18 exon skipping to promote immune escape from gallbladder cancer. Furthermore, H3K36me3 regulated IL‐18 exon skipping by recruiting PTBP3 via MRG15 and coupled IL‐18 transcription and alternative splicing. Mechanistically, ΔIL‐18 decreased FBXO38 transcription levels in CD8+T cells to reduce FBXO38‐mediated degradation of PD‐1. During splicing, the hnRNPL bound to H3K36 methyltransferases SETD2 and this manner interfered the binding of PTBP3 to the IL‐18 pre‐mRNA.

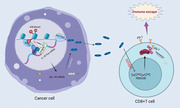

## 1 Introduction

Although gallbladder cancer is a highly malignant tumor of the biliary system, it is often diagnosed at a late stage due to the lack of typical clinical symptoms and early diagnostic tools.^[^ ^1^, ^2^ ^]^ Currently, surgical resection is the accepted treatment for gallbladder cancer, but few patients are able to undergo surgery, and the efficacy of various adjuvant treatment modalities is limited.^[^ ^3^, ^4^ ^]^ Therefore, it is necessary to more thoroughly elucidate the pathogenesis of gallbladder cancer and identify effective therapeutic targets.

The transfer of genetic information from DNA to mature mRNA in higher eukaryotes often requires pre‐mRNA splicing.^[^ ^5^ ^]^ Most intron‐containing genes undergo simple intron splicing and exon joining, a process known as constitutive splicing.^[^ ^6^ ^]^ Alternative splicing provides additional modes of gene regulation that can significantly increase transcript numbers and enhance the structural and functional diversity of proteins.^[^ ^7^, ^8^ ^]^ Although the different splice isoforms of many proteins are beneficial under normal conditions, alternative splicing under pathological conditions can lead to detrimental effects.^[^ ^9^ ^]^ For example, in tumor tissues, extensive alternative splicing abnormalities have been shown to contribute to tumor progression.^[^ ^10^ ^]^ There are five major modes of alternative splicing, of which exon skipping is the most common. Alternative splicing‐associated RNA‐binding proteins (RBPs) play an important role in regulating alternative splicing events during tumorigenesis.^[^ ^11^ ^]^ For example, DDX17 was shown to regulate alternative splicing to generate an oncogenic isoform of PXN‐AS1 that promotes HCC metastasis.^[^ ^12^ ^]^ In addition, our previous study showed that a non‐POU domain‐containing octamer‐binding protein promotes gallbladder cancer cell proliferation by enhancing the oncogenic RNA splicing of DLG1.^[^ ^1^ ^]^ However, we are still far from a complete understanding of the alternative splicing abnormalities in gallbladder cancer.

PTBP3, also known as “regulator of differentiation 1”, is a member of the polypyrimidine bundle binding protein family.^[^ ^13^ ^]^ PTBP3 plays important roles in RNA splicing, translational activation, and mRNA stabilization.^[^ ^14^ ^]^ Recent studies have shown that PTBP3 acts as an oncogene and promotes the progression of hepatocellular carcinoma, gastric cancer, and colorectal cancer.^[^ ^15^, ^16^, ^17^ ^]^ Here, using bioinformatics screening, we discovered that PTBP3 was highly expressed in gallbladder cancer, but had no significant effect on the biological behavior of tumor cells. With the help of multi‐omics, PTBP3 was identified to promote exon skipping of IL‐18, resulting in the generation of ΔIL‐18, which is specifically expressed in tumors. Further, we found that PTBP3 promoted immune escape in gallbladder cancer via ΔIL‐18, which decreased FBXO38 transcription levels in CD8+T cells to reduce FBXO38‐mediated degradation of PD‐1. We also found that H3K36me3 can regulate IL‐18 exon skipping by recruiting PTBP3 via MRG15, coupling IL‐18 transcription and alternative splicing. During this process, hnRNPL binds to the H3K36 methyltransferase SETD2, which interferes with the binding of PTBP3 to IL‐18 pre‐mRNA. Together, our findings reveal that PTBP3 modulates exon skipping of IL‐18 to promote immune escape in gallbladder cancer, providing a new perspective for gallbladder cancer immunotherapy by blocking ΔIL‐18 production.

## 2 Results

### 2.1 Over‐Expression of the Splicing factor PTBP3 is Significantly Associated with Poor Prognosis in Gallbladder Cancer

**Figure 1:**
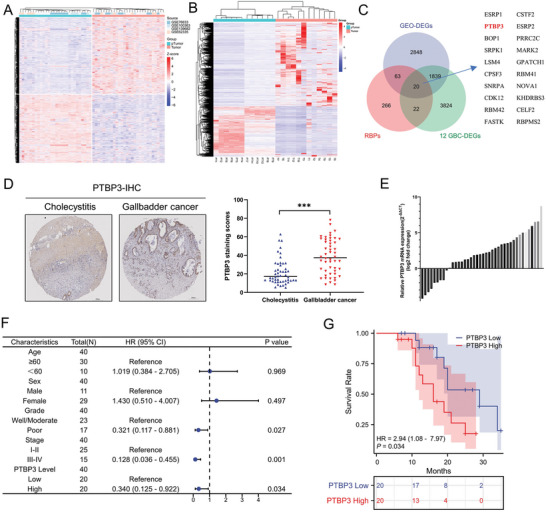
Over‐expression of the splicing factor PTBP3 is significantly associated with poor prognosis in gallbladder cancer. A) Heatmap of differentially expressed genes obtained by integrating four GEO datasets (GSE76633, GSE100363, GSE139682, and GSE62335). B) Heatmap of differentially expressed genes from 12 pairs of gallbladder cancer mRNA sequencing. C) The panel on the left is Venn plots based on the GEO database of differentially expressed genes, 12 pairs of differentially expressed genes made from the gallbladder cancer transcriptome and alternative splicing factor datasets; the panel on the right is a list of 20 intersecting genes. D) The left panel is a representative image from tissue microarray including cholecystitis and gallbladder cancer tissue immunohistochemistry results using anti‐PTBP3 antibody, and the right panel is the immunohistochemistry score. E) Slope chart of PTBP3 mRNA expression in 40 pairs of gallbladder cancer tissues. F) Forest plot of 40 gallbladder cancer patients analyzed by clustering based on high and low PTBP3 expression levels. G) Kaplan‐Meier overall survival curve of gallbladder cancer patients according to PTBP3 mRNA expression level showed that patients with high expression of PTBP3 had a worse prognosis.

To identify potential alternative splicing factors that promote gallbladder cancer progression, we analyzed the splicing factor dataset (Table S4, Supporting Information), integrated GEO gallbladder cancer dataset (GSE76633, GSE100363, GSE139682, and GSE62335)^[^ ^19^, ^20^, ^21^, ^22^ ^]^ and the mRNA‐seq data of 12 patients with gallbladder cancer (**Figure** **1**A,B). The Venn diagram shows that 20 splicing factors were aberrantly expressed in gallbladder cancer at the mRNA level (Figure 1C). We decided to focus on PTBP3 because it was so markedly overexpressed, yet little is known about its function in gallbladder cancer. Next, we analyzed PTBP3 protein expression levels in tissue microarrays containing 50 gallbladder cancer and 50 cholecystitis tissue samples using IHC. As shown in Figure 1D, PTBP3 protein expression was higher in gallbladder cancer tissues than in cholecystitis tissues. We also examined PTBP3 protein levels in the 12 pairs of gallbladder cancer and para‐cancerous tissues that we had previously used for mRNA sequencing. The western blotting results revealed higher expression levels of PTBP3 in the cancer tissues than in their matched controls (Figure S1A, Supporting Information). To investigate the association between PTBP3 and clinical prognosis, we detected mRNA expression in 40 pairs of gallbladder cancer tissue samples using qPCR, and subsequently divided the samples equally into two groups based the logFold change values (low/high) (Figure 1E). Through data analysis, we found that high PTBP3 expression was correlated with the histopathological grading and TNM stage of the patients (Figure 1F). Furthermore, Kaplan‐Meier curve analysis showed that patients with high PTBP3 expression had worse overall survival rates (Figure 1G). These results suggest that PTBP3 plays an important role in gallbladder cancer progression.

### 2.2 PTBP3 Promotes IL‐18 Exon Skipping in Gallbladder Cancer

**Figure 2:**
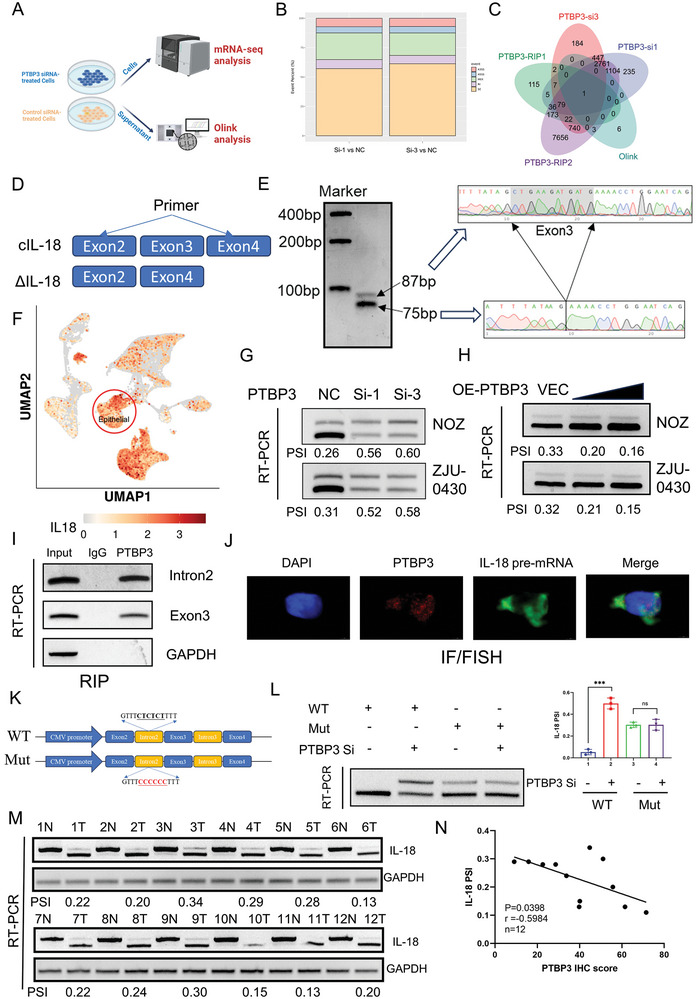
PTBP3 promotes IL‐18 exon skipping in gallbladder cancer. A) Schematic representation of the multi‐omics analysis of NOZ cells. In brief, PTBP3 was knocked down in NOZ cells, while cell supernatants were later collected for Olink proteomics assay and extracted cell total RNA was collected for mRNA sequencing. B) Alternative splicing differential event analysis based on transcriptome sequencing after PTBP3 knockdown in NOZ cells. C) Venn diagram analysis combining differential proteins detected by Olink proteomics, differential exon skipping events in mRNA‐seq, mRNAs bound by PTBP3 analyzed by our RIP‐seq data and previously reported RIP‐seq data. D) Schematic of the two IL‐18 transcripts and primer design positions. “cIL‐18″ stands for classic IL‐18. E) The left panel represents the DNA electrophoresis of the two isoforms of IL‐18 detected by applying PCR to untreated NOZ cells; the right panel represents the results of sanger sequencing of two bands. The two bands differ for exon 6. F) Gallbladder cancer single cell transcriptome analysis showed that gallbladder cancer cell could express IL‐18 using UMAP plots. G) RT‐PCR analysis IL‐18 isoform under PTBP3 knockdown. PSI = splice_in gray value / (splice_in gray value+splice_out gray value). H) RT‐PCR analysis of IL‐18 isoform under PTBP3 overexpression. I) RT‐PCR analysis of RIP results using anti‐PTBP3 antibody to test for Intron2 and Exon3 level. J) FISH/IF assay to analyze IL‐18 pre‐mRNA and PTBP3 localization in NOZ. K) Schematic diagram of the IL‐18 minigene constructs. L) Splicing analysis of IL‐18 minigene reporter in NOZ cells (*P<0.05, **P<0.01, Student's t‐test). Data are expressed as mean±SD, n = 3. M) RT‐PCR analysis of two isoforms of IL‐18 in 12 pairs of gallbladder cancer tissues and paracancerous tissues. N) Correlation analysis of IL‐18 PSI value and PTBP3 expression shows a negative correlation between the two by Spearman anaysis.

To investigate the role of PTBP3 in gallbladder carcinogenesis, we knocked down PTBP3 using siRNAs in two gallbladder cancer cell lines with high PTBP3 expression (Figure S1B–D, Supporting Information). Cell proliferation and transwell assays indicated that PTBP3 knockdown in gallbladder cancer cells did not significantly affect their proliferation, invasion, or migration ability (Figure S1E–G, Supporting Information). Cytokines are important means of communication between immune and non‐immune cells and tissues.^[^ ^23^ ^]^ Therefore, we hypothesized that PTBP3 may indirectly influence gallbladder cancer progression through other cells in the microenvironment. Following siRNA‐mediated suppression of PTBP3 in NOZ cells, we collected cell supernatants for Olink proteomics, and extracted total cellular RNA for mRNA sequencing (**Figure** **2**A). Olink proteomics results showed that PTBP3 knockdown significantly downregulated the expression of six cytokines in the supernatants of NOZ cells (Figure S2A, Supporting Information). In addition, as PTBP3 is an alternative splicing factor, its knockdown resulted in changes in alternative splicing events, which were dominated by exon skipping (Figure 2B; Figure S2B, Supporting Information). To investigate the RNAs that bind to PTBP3, RIP‐seq was performed using an anti‐PTBP3 antibody. This revealed that the RNAs bound to PTBP3 were predominantly mRNAs, and that PTBP3 bound predominantly to exons and introns (Figure S2C,D, Supporting Information). Next, we compared the results of the Olink proteomics experiment, the mRNA‐seq analysis, our RIP‐seq experiment, and previously reported RIP‐seq data to explore the downstream genes potentially regulated by PTBP3.^[^ ^24^ ^]^ Integration of the results revealed that IL‐18 was the only gene common to all five datasets (Figure 2C). We applied ELISA an qPCR assay to verify that PTBP3 promoted IL‐18 expression at both protein and mRNA levels (Figure S2E–H, Supporting Information).

Based on the results of the alternative splicing event analysis of our mRNA‐seq experiment, we found that IL‐18 exists in two distinct transcripts: NM_0 013 86420.1, and NM_0 012 43211.2. NM_0 012 43211.2 (known as ΔIL‐18) lacks an in‐frame coding exon3 (12 bp) compared to classic NM_0 013 86420.1 (known as cIL‐18), meaning that the ΔIL‐18 protein lacks four amino acids compared to full‐length cIL‐18 (Figure 2D). As the two isoforms are similar sizes, it would be difficult to distinguish between the two proteins by western blotting. Accordingly, we designed specific PCR primers to validate our results. DNA electrophoresis following PCR showed the presence of two bands at 87 and 75 bp, and subsequent Sanger sequencing revealed that the difference between the PCR products corresponded to exon3 of IL‐18 (Figure 2E). In addition, we analyzed gallbladder cancer single‐cell sequencing data,^[^ ^25^ ^]^ and found that, although IL‐18 expression was detected in gallbladder cancer cells, there was no way of distinguishing between expression of cIL‐18 or ΔIL‐18 (Figure 2F). Next, we discovered that knockdown of PTBP3 could inhibit exon skipping of IL‐18, whereas overexpression of PTBP3 promoted exon skipping of IL‐18 (Figure 2G,H). Using the RIP assay, we verified that PTBP3 could bind to the pre‐mRNA of IL‐18 (Figure 2I), and FISH/IF experiments also confirmed that PTBP3 colocalized with IL‐18 pre‐mRNA (Figure 2J). It has been shown that CUCUCU is the most enriched sequence observed in the PTBP3 binding sites of pre‐mRNAs.^[^ ^26^ ^]^ Accordingly, we identified a possible binding site on intron2 of IL‐18 and constructed a corresponding mutant for RNA pulldown, which revealed that PTBP3 bound to IL‐18 pre‐mRNA through this site (Figure 2I). Furthermore, we used the IL‐18 minigene reporter to validate the regulation of IL‐18 splicing by PTBP3 (Figure 2K). The results showed that PTBP3‐mediated IL‐18 splicing is dependent on this splice site (Figure 2L). A previous study reported that ΔIL‐18 was expressed in ovarian cancer but not in normal ovarian epithelium.^[^ ^27^ ^]^ We therefore examined ΔIL‐18 and cIL‐18 levels in 12 pairs of gallbladder cancer tissues and their matched control tissues and found that ΔIL‐18 was detected only in the cancer tissues. In addition, we found that ΔIL‐18 was only detected in gallbladder cancer tissues and cells, and not in normal tissues and cell (Figure 2M; Figure S2J, Supporting Information). Moreover, the PSI values of IL‐18 in the 12 gallbladder cancer samples were negatively correlated with the expression level of PTBP3 protein, consistent with the finding that PTBP3 promotes exon skipping of IL‐18 (Figure 2N). In conclusion, our results show that PTBP3 promotes exon skipping of IL‐18 in gallbladder cancer.

### 2.3 PTBP3 Promotes Gallbladder Cancer Immune Escape through ΔIL‐18

**Figure 3:**
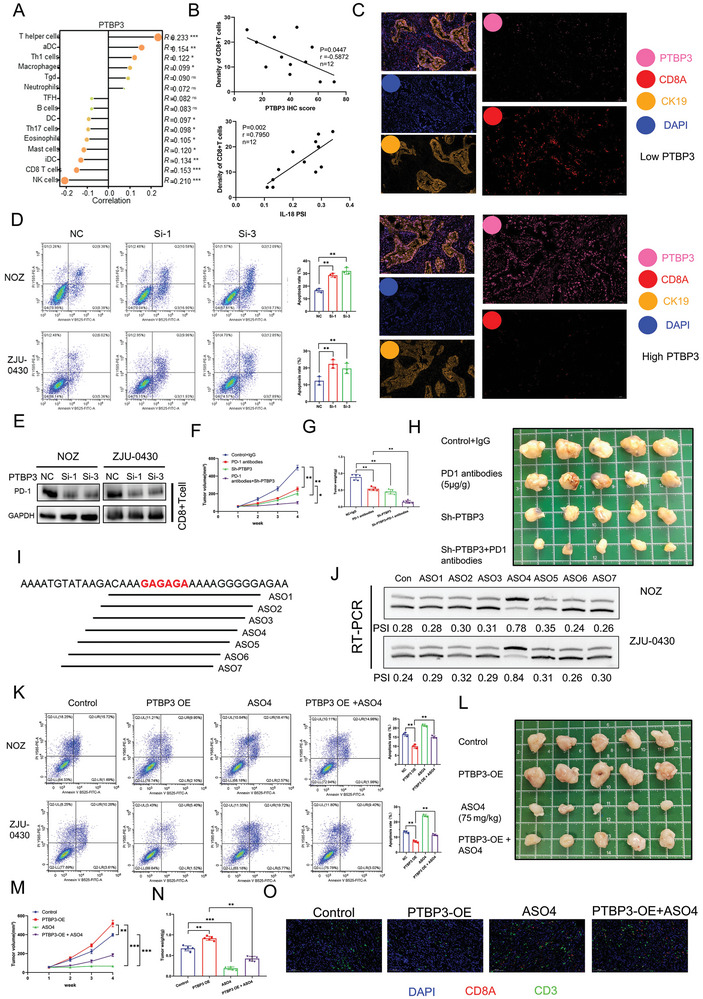
PTBP3 promotes gallbladder cancer immune escape through ΔIL‐18. A) Lollipop Chart of analysis of immune cell infiltration associated with PTBP3 expression based on 12 pairs of GBC mRNA‐seq data. B) The panel in the upper position is a scatter plot of correlation between PTBP3 immunohistochemical score and CD8+T cell infiltration by Spearman analysis; the panel in the bottom position is a scatter plot of the correlation between IL‐18 PSI value and CD8+T cell infiltration by Spearman analysis. C) Labeling of gallbladder cancer tissues with KI67, PTBP3 and CD8A using multiplex immunohistochemistry. D) Evaluation of tumor cell killing capacity of T cells after PTBP3 knockdown in tumor cells using flow cytometry (CD8+T cells and tumor cells co‐cultured at a ratio of 1:1 for 48 h). In brief, the treated tumor cells were spread in a 6‐well plate, and after the cells were completely attached to the wall, a 1:1 quantity of CD8+T cells was added. At 48 hours later, the residual liquid was removed from each well, and cells were washed with PBS followed by flow cytometry detection. E) Effect of PTBP3 knockdown in GBC cells on PD‐1 expression in CD8+T cells using western blotting in a co‐culture system (CD8+T cells and tumor cells co‐cultured at a ratio of 1:1 for 48 h). F) Measurement of subcutaneous tumor growth volume in HuPBMC NOG mice. G) Measurement of tumor weight in HuPBMC NOG mice. H) Illustration of a subcutaneous tumor in HuPBMC NOG mice. I) Schematic diagram of a series of ASO drugs designed to target the splicing site of IL‐18. J) Transfection of ASO in tumor cells followed by RT‐PCR assay to test the inhibitory efficiency of the ASO drug on IL‐18. K) Evaluation of tumor cell killing capacity of T cells after PTBP3 overexpression or ASO4 treatment in tumor cells using flow cytometry (CD8+T cells and tumor cells co‐cultured at a ratio of 1:1 for 48 h). L) Illustration of a subcutaneous tumor in HuPBMC NOG mice. M) Measurement of subcutaneous tumor growth volume in HuPBMC NOG mice. N) Measurement of tumor weight in HuPBMC NOG mice. O) Labeling of CD8A and CD3 in subcutaneous tumors using multiplex immunohistochemistry. Statistical tests involved: *P < 0.05, **P < 0.01, Student's *t*‐test; Data are expressed as mean±SD, n = 3.

Based on the mRNA‐seq data from our 12 patients with gallbladder cancer, we used Spearman's analysis to explore the relationship between PTBP3 and immune cell infiltration. As shown in **Figure** **3**A, NK and CD8+T cells were significantly negatively correlated with PTBP3 expression. CD8+T cells, which are important immune cells involved in tumorigenesis and development, are known to play a role in tumor cell immune escape.^[^ ^28^, ^29^ ^]^ We found that the expression of PTBP3 was negatively correlated with CD8+T cell infiltration, whereas the IL‐18 PSI value was positively correlated with CD8+T cell infiltration (Figure 3B,C). Based on these results, we used a tumor‐ and CD8+T‐cell co‐culture system to show that PTBP3 knockdown in tumor cells enhanced the anti‐tumor effects of CD8+T cells (Figure 3D; Figure S3A, Supporting Information). The results of ELISA assays indicated that CD8+T cells produced more granzyme B and IFNγ upon co‐culturing with PTBP3 knockdown NOZ and ZJU‐0430 cells (Figure S3B,C, Supporting Information). Dysregulation of immune checkpoints plays an important role in immune evasion in many malignant tumors.^[^ ^30^ ^]^ Among them, disruption of the PD‐1 /PD‐L1 axis was shown to be significantly associated with tumor progression.^[^ ^31^ ^]^ The results of the mRNA‐seq analysis demonstrated that the expression of PTBP3 did not significantly correlate with PD‐L1 expression, but did positively correlate with PD‐1 expression in the 12 paired gallbladder cancer samples (Figure S3D, Supporting Information). Furthermore, western blotting showed that knockdown of PTBP3 in gallbladder cancer cells in the CD8+T cell co‐culture system had no significant effect on PD‐L1 expression, but significantly reduced PD‐1 expression in the CD8+T cells (Figure 3E; Figure S3E, Supporting Information). Next, we constructed a HuPBMC mouse model to investigate the role of PTBP3 in the anti‐tumor function and immunotherapy sensitivity of CD8+T cells in gallbladder in vivo (Figure S3F, Supporting Information). We verified the HuPBMC mouse model using flow cytometry, which confirmed that the percentage of human CD45+ cells in the mice was greater than 60% (Figure S3G, Supporting Information). The in vivo experiment showed that PTBP3 knockdown significantly inhibited HuPBMC tumor growth and improved immunotherapeutic sensitivity in gallbladder cancer, as measured by subcutaneous tumor volume and mass (Figure 3F–H; Figure S3H, Supporting Information). In addition, we performed mIHC of subcutaneous tumors and found more infiltrating CD8+T cells in the PTBP3 knockdown group than in the control group (Figure S3I, Supporting Information). In contrast, there was no significant effect on tumor growth after PTBP3 knockdown in the NOG mouse subcutaneous tumor model, suggesting that the anti‐tumor effect mediated by PTBP3 knockdown was CD8+T cell‐dependent (Figure S3J–M, Supporting Information).

Antisense oligonucleotides (ASOs) are powerful therapeutic tools that bind to specific RNA target sequences through complementary base pairing, leading to RNA degradation, changes in RNA splicing, translation inhibition, or disruption of RNA‐protein interactions.^[^ ^32^ ^]^ Therefore, we designed and synthesized a series of 2′‐Omethoxyethy‐modified ASOs to inhibit ΔIL‐18 production in tumor cells by blocking the splicing site of IL‐18 (Figure 3I). One of the ASOs, ASO4, was particularly successful at significantly reducing the production of ΔIL‐18 (Figure 3J). To investigate whether PTBP3 mediates gallbladder cancer immune escape via ΔIL‐18, we overexpressed PTBP3 in tumor cells and treated them with ASO4 to reduce ΔIL‐18 production. Flow cytometric analysis showed that ASO4 partially reversed the inhibition of apoptosis caused by PTBP3 overexpression (Figure 3K). ELISA assay results also showed that ASO treatment was able to rescue the decrease in granzyme B and IFNγ secretion by CD8+ T cells caused by PTBP3 overexpression (Figure S3N,O, Supporting Information). In addition, western blotting showed that ASO4 treatment reduced PD‐1 expression in CD8+T cells, and also reduced the increase in PD‐1 expression induced by PTBP3 (Figure S3P, Supporting Information). In vivo experiments using the HuPBMC mouse model also confirmed that ASO4 reduced tumor progression caused by PTBP3 overexpression and showed significant anti‐tumor effects (Figure 3L–N; Figure S3Q, Supporting Information). mIHC showed that ASO4 treatment increased CD8+T cell infiltration within the tumor, consistent with the anti‐tumor effect being via CD8+T‐cell function (Figure 3O). In terms of drug safety, no significant pathological changes were observed in the major organs (heart, liver, kidneys, and lungs) of mice treated with ASO4 (Figure S3R, Supporting Information). Taken together, PTBP3‐mediated pro‐tumorigenic effects are CD8+T cell‐dependent and ASO4 may have potential value in the treatment of gallbladder cancer.

### 2.4 ΔIL‐18 Reduces PD‐1 Ubiquitination by Inhibiting FBXO38 Transcription

**Figure 4:**
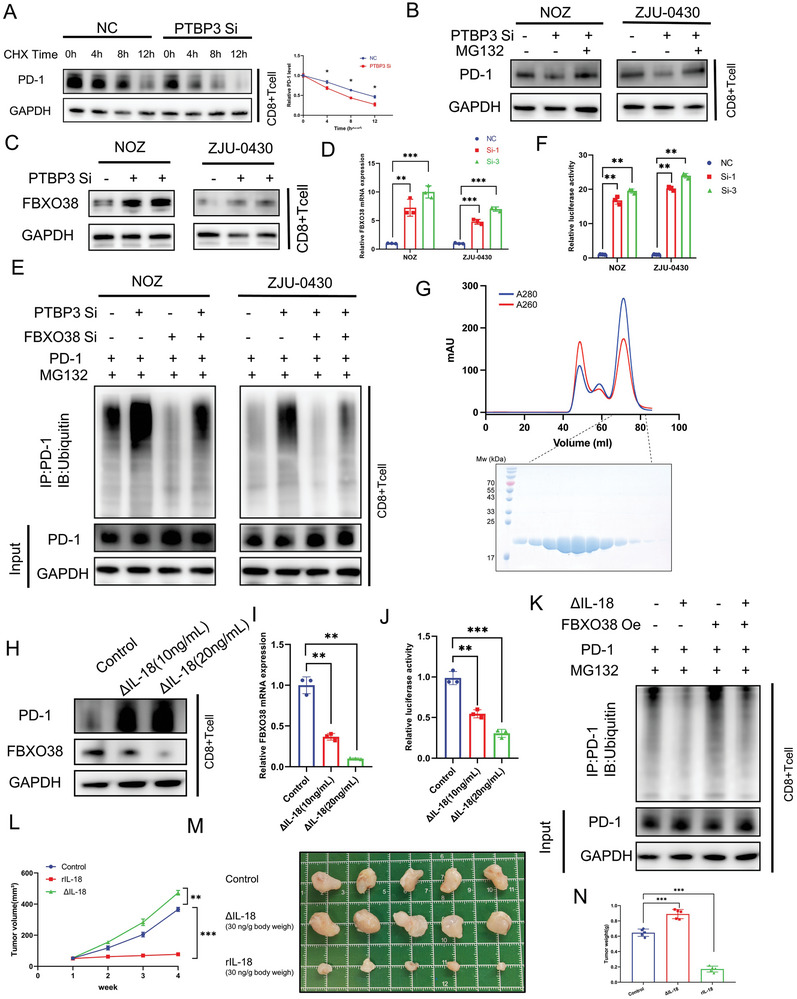
ΔIL‐18 reduces PD‐1 ubiquitination by inhibiting FBXO38 transcription. A) CD8+T cells with tumor cell (with or without PTBP3 knockdown) supernatants were treated with 60 µg ml^−1^ CHX, then the proteins were extracted according to the time point for detecting PD‐1 levels using western blotting. B) Levels of PD‐1 in CD8+T cells with tumor cell (with or without PTBP3 knockdown) supernatants were detected by western blotting after 12 h treatment with MG132 (20 µM). C) Levels of FBXO38 in CD8+T cells with tumor cell (with or without PTBP3 knockdown) supernatants were detected by western blotting. D) Levels of FBXO38 in CD8+T cells with tumor cell (with or without PTBP3 knockdown) supernatants were detected by qPCR. E) Levels of ubiquitination of PD‐1 in CD8+T cells with tumor cell (with or without PTBP3 knockdown) supernatants were detected by western blotting. F) Levels of transcriptional activity of FBXO38 in CD8+T cells with tumor cell (with or without PTBP3 knockdown) supernatants were detected by luciferase reporter assay. G) The up panel, the absorbent peak graph at 260 and 280 nm of ΔIL18 purified by size‐exclusive chromatography. The down panel, the SDS‐page stained by Coomassie blue, showed the purified ΔIL18 protein. H) PD‐1 and FBXO38 expression were detected by western blotting after treatment of CD8+T cells with two concentrations of ΔIL‐18. I) FBXO38 expression were detected by qPCR after treatment of CD8+T cells with two concentrations of ΔIL‐18. J) Levels of transcriptional activity of FBXO38 in CD8+T cells were detected by luciferase reporter assay. K) Levels of ubiquitination of PD‐1 in CD8+T cells with ΔIL‐18 treatment were detected by western blotting. L) Measurement of subcutaneous tumor growth volume in HuPBMC NOG mice. M) Illustration of a subcutaneous tumor in HuPBMC NOG mice. N) Measurement of tumor weight in HuPBMC NOG mice. Statistical tests involved: *P < 0.05, **P < 0.01, Student's *t*‐test; Data are expressed as mean±SD, n = 3.

IL‐18 is a myeloid leukocyte inflammatory mediator whose primary function is to induce IFNγ secretion by T cells and NK cells.^[^ ^33^ ^]^ In a mouse model, IL‐18 expression enhanced T cell proliferation, cytotoxicity, and persistence, thereby inhibiting tumor progression.^[^ ^34^ ^]^ In vitro experiments showed that r IL‐18 did not have an obvious effect on the proliferative capacity of gallbladder tumor cells (Figure S4A, Supporting Information). Nevertheless, T cells pre‐treated with rIL‐18 displayed significantly enhanced killing effects on gallbladder cancer cells (Figure S4B, Supporting Information). In addition, IFNγ ELISA demonstrated that rIL‐18 enhanced IFNγ secretion from CD8+T cells (Figure S4C, Supporting Information). These results reveal that classic IL‐18 enhances the anti‐tumor effect of CD8+T cells in gallbladder cancer. To explore the mechanism by which PTBP3 affects PD‐1 expression in CD8+T cells, we first used qPCR to detect PD‐1 mRNA, but found that PTBP3 knockdown did not affect PD‐1 mRNA levels (Figure S4D, Supporting Information). This indicates that PTBP3 may influence PD‐1 expression at the protein level. Further examination revealed that PD‐1 protein stability was decreased after PTBP3 knockdown, whereas PTBP3 overexpression enhanced PD‐1 protein stability (**Figure** **4**A; Figure S4E, Supporting Information). Protein stability can be regulated through the proteasomal or lysosomal degradation pathways. We found that treatment with the proteasomal inhibitor MG132 inhibited the decrease in PD‐1 protein levels caused by PTBP3 knockdown, implicating the proteasomal degradation pathway (Figure 4B; Figure S4F, Supporting Information).^[^ ^35^ ^]^ It has been reported that FBXO38 mediates PD‐1 ubiquitination and regulates the anti‐tumor function of T cells.^[^ ^36^ ^]^ Interestingly, PTBP3 knockdown significantly upregulated FBXO38 at both the RNA and protein levels (Figure 4C,D). We also confirmed that FBXO38 regulates PD‐1 ubiquitination to modulate its protein expression in gallbladder cancer cells (Figure S4G,H, Supporting Information). Further experiments revealed that PTBP3 knockdown or ASO4 treatment increased PD‐1 ubiquitin‐mediated degradation, whereas FBXO38 knockdown decreased the ubiquitin‐mediated degradation of PD‐1 (Figure 4E; Figure S4I, Supporting Information). Luciferase assays demonstrated that PTBP3 affected FBXO38 expression by reducing its transcription (Figure 4F; Figure S4J, Supporting Information). As all our previous experiments have been based on manipulating PTBP3 expression or ASO4 treatment, we decided to directly investigate the effect of ΔIL‐18 on CD8+T cells by purifying ΔIL‐18 (Figure 4G). Treatment of CD8+T cells with ΔIL‐18 increased the expression and stability of PD‐1, but decreased the expression of FBXO38 by decreasing its transcription level (Figure 4H–J; Figure S4K,L, Supporting Information). Further experiments showed that ΔIL‐18 reduced PD‐1 ubiquitination through FBXO38 (Figure 4K). In addition, by employing the HuPBMC model, we found that rIL‐18 inhibited the growth of subcutaneous gallbladder cancer tumors in vivo, whereas ΔIL‐18 significantly promoted their growth (Figure 4L–N; Figure S4M, Supporting Information). To summarize, PTBP3‐mediated exon skipping of IL‐18 dramatically changes its function in gallbladder cancer; while cIL‐18 inhibits tumor growth, ΔIL‐18 down‐regulates FBXO38 transcription levels in CD8+T cells to reduce PD‐1 ubiquitin‐mediated degradation, thereby promoting gallbladder cancer immune escape.

### 2.5 The Histone Modification H3K36me3 Couples IL‐18 Transcription and Alternative Splicing

**Figure 5:**
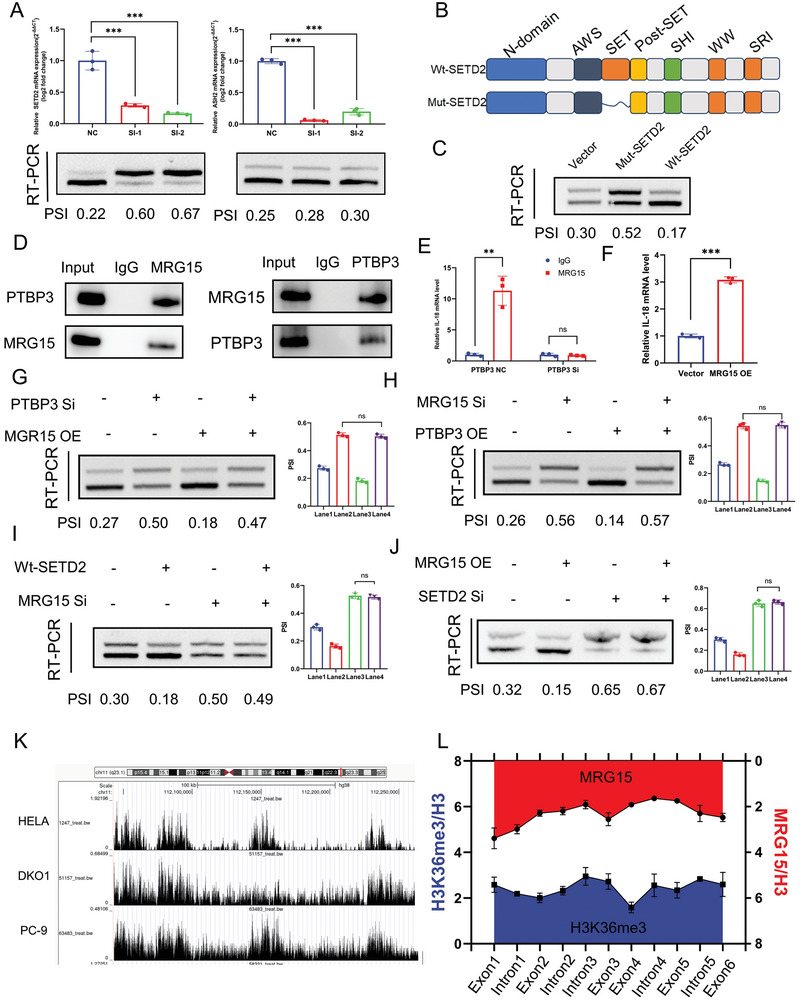
The histone modification H3K36me3 couples IL‐18 transcription and alternative splicing. A) The left panel showed the results of validation of the effect of knockdown of H3K36 methyltransferase SETD2 on exon skipping of IL‐18 using RT‐PCR assay in NOZ; the right panel showed validation of the effect of knockdown of H3K4 methyltransferase ASH2 on exon skipping of IL‐18 using RT‐PCR assay in NOZ. B) Schematic representation of the SETD2 domain. C) RT‐PCR experiments were performed after transfection of NOZ with Wt‐SETD2 and Mut‐SETD2 to determine their effects on IL‐18 exon skipping. D) Western blotting results after immunoprecipitation experiments using MRG15 and PTBP3 antibodies, respectively. E) The PTBP3 normal control group and PTBP3 siRNA group were subjected to RIP experiments with MRG15 antibody to investigate the binding of IL‐18 by MRG15, respectively. F) RIP experiments using PTBP3 antibody after overexpression of MRG15 to probe the binding of PTBP3 to IL‐18. G) RT‐PCR experiments were performed after knockdown of PTBP3 and overexpression of MRG15, respectively H) RT‐PCR experiments were performed after knockdown of MRG15 and overexpression of PTBP3, respectively. I) RT‐PCR experiments were performed after knockdown of MRG15 and overexpression of Wt‐SETD2, respectively. J) RT‐PCR experiments were performed after knockdown of SETD2 and overexpression of MRG15, respectively. K) Analysis of H3K36me3 peaks on IL‐18 in multiple tumor cells using Cistrome Data Browser. L) Chromatin immunoprecipitation of H3K36me3 and MRG15 along IL‐18. Statistical tests involved: *P < 0.05, **P < 0.01, Student's *t*‐test; Data are expressed as mean±SD, n = 3.

Pre‐mRNA splicing occurs largely in a co‐transcriptional manner and is influenced by RNA polymerase II extension rates and chromatin remodeling.^[^ ^37^ ^]^ H3K36me3 and H3K4me3 are two major histone modifications involved in alternative splicing; therefore, we knocked down their specific methyltransferases and examined the effects on the alternative splicing of IL‐18 using RT‐PCR.^[^ ^38^ ^]^ Exon skipping of IL‐18 was significantly inhibited only after knockdown of the H3K36 methyltransferase SETD2 (**Figure** **5**A; Figure S5A, Supporting Information). The SET domain is responsible for catalyzing trimethylation of H3K36; therefore we constructed a mutant SETD2 plasmid lacking this domain (Figure 5B; Figure S5B, Supporting Information).^[^ ^39^ ^]^ This allowed us to demonstrate that, while overexpression of Wt‐SETD2 promoted IL‐18 exon skipping, Mut‐SETD2 inhibited exon skipping to some degree (Figure 5C). To elucidate the molecular mechanisms by which histone modifications affect alternative splicing, we focused on the histone tail‐binding protein MRG15, which specifically binds to H3K36me3.^[^ ^40^ ^]^ We found that exon skipping of IL‐18 was significantly inhibited after MRG15 knockdown (Figure S5C, Supporting Information). Immunoprecipitation revealed that MRG15 could bind to PTBP3 (Figure 5D). In addition, RIP using an anti‐MRG15 antibody indicated that MRG15 could bind to IL‐18 pre‐mRNA, and that this interaction was significantly inhibited after PTBP3 knockdown (Figure 5E). In contrast, MRG15 overexpression significantly enhanced the binding of PTBP3 to IL‐18 pre‐mRNA (Figure 5F). Overexpression of MRG15 promoted exon skipping of IL‐18, whereas PTBP3 knockdown attenuated IL‐18 exon skipping promoted by MRG15 (Figure 5G). Moreover, knockdown of MRG15 diminished the IL‐18 exon skipping promoted by PTBP3 overexpression (Figure 5H). These results suggest that PTBP3‐mediated exon skipping of IL‐18 is dependent on MGR15. Further, IL‐18 exon skipping induced by overexpression of SETD2 was inhibited by MRG15 knockdown (Figure 5I). In addition, knockdown of SETD2 attenuated IL‐18 exon skipping promoted by MRG15 overexpression (Figure 5J). We tentatively conclude that H3K36me3 recruits PTBP3 via MRG15, which in turn regulates the IL‐18 exon skipping process. Furthermore, we found multiple peaks of H3K36me3 on IL‐18 DNA in multiple tumor cell lines using the Cistrome Data Browser (http://cistrome.org/db/#/) (Figure 5K). Using ChIP‐qPCR, we found that H3K36me3 and MRG15 were enriched across the IL‐18 gene in NOZ cells (Figure 5L; Figure S5D, Supporting Information). Taken together, these results suggest that H3K36me3 couples IL‐18 transcription and alternative splicing.

### 2.6 SETD2/hnRNPL Interferes with PTBP3 Binding to IL‐18 pre‐mRNA

**Figure 6:**
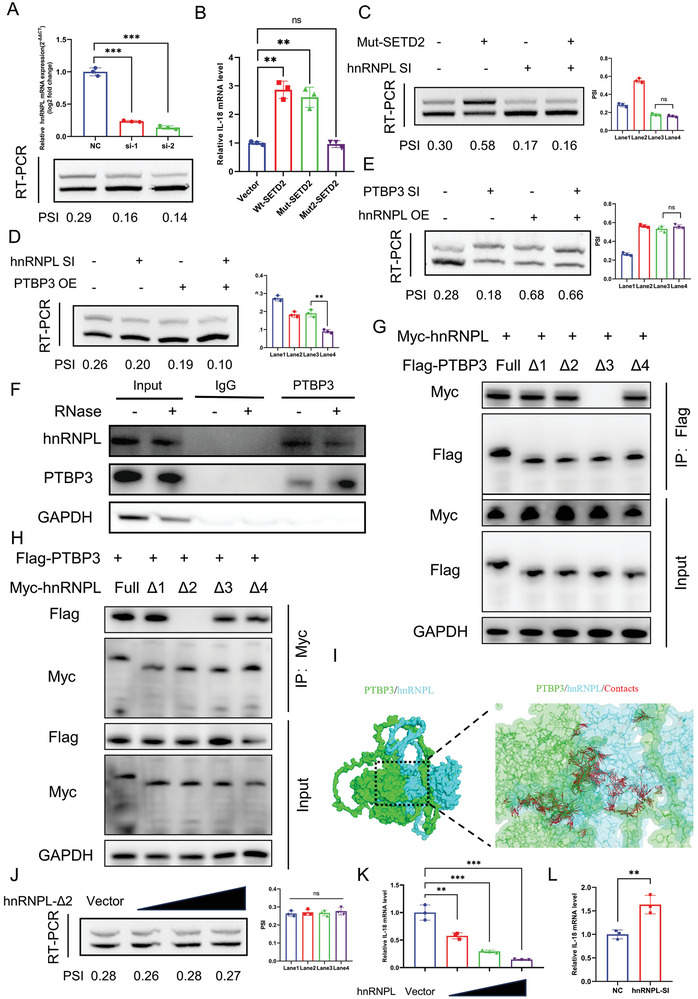
SETD2/hnRNPL interferes with PTBP3 binding to IL‐18 pre‐mRNA. A) Validation of the effect of knockdown of hnRNPL on exon skipping of IL‐18 using RT‐PCR assay in NOZ. B) RIP assay with anti‐hnRNPL antibody after overexpression of Wt‐SETD2∖Mut‐SETD2∖Mut2‐SETD2. C) RT‐PCR experiments were performed after knockdown of hnRNPL and overexpression of Mut‐SETD2, respectively. D) RT‐PCR experiments were performed after knockdown of hnRNPL and overexpression of PTBP3, respectively. E) RT‐PCR experiments were performed after knockdown of PTBP3 and overexpression of hnRNPL, respectively. F) Validation of PTBP3 binding to hnRNPL using western blotting with or without RNase. G) Myc‐tagged hnRNPL plasmid and different Flag‐tagged deletion mutant PTBP3 plasmid were transfected into 293T cells and performed IP assays. H) Flag‐tagged PTBP3 plasmid and different Myc‐tagged deletion mutant hnRNPL plasmid were transfected into 293T cells and performed IP assays. I) Molecular docking results of PTBP3 and hnRNPL presented by PyMol software. J) RT‐PCR experiments were performed after overexpression of hnRNPL‐Δ2 under transfection with different plasmid concentrations. K) RIP assay with anti‐PTBP3 antibody after overexpression of hnRNPL under transfection with different plasmid concentrations. L) RIP assay with anti‐PTBP3 antibody after knockdown of hnRNPL. Statistical tests involved: *P < 0.05, **P < 0.01, Student's *t>*‐test; Data are expressed as mean±SD, n = 3.

In the previous section, we found that IL‐18 exon skipping was inhibited after overexpression of Mut‐SETD2 relative to the vector control group in NOZ cells (Figure 5B,C). A previous study indicated that interactions between the SHI domain of SETD2 and hnRNPL regulates a range of gene transcription and alternative splicing events.^[^ ^41^ ^]^ We found that knockdown of hnRNPL enhanced exon skipping of IL‐18, whereas overexpression of hnRNPL inhibited exon skipping of IL‐18 (**Figure** **6**A; Figure S5E, Supporting Information). It has been found that hnRNPL binds to the CA repeat sequence of RNA.^[^ ^42^ ^]^ Accordingly, we identified a possible binding site on intron2 of IL‐18 and constructed a corresponding mutant for RNA pull‐down experiments, which revealed that hnRNPL bound to IL‐18 pre‐mRNA through this site (Figure S5F). This also indicated that hnRNPL could bind directly to the IL‐18 pre‐mRNA. Next, we constructed the SHI domain‐deletion plasmid, Mut2‐SETD2. Using RIP with anti‐hnRNPL antibodies, we found that both Wt‐SETD2 and Mut‐SETD2 enhanced the binding of hnRNPL to the IL‐18 pre‐mRNA, whereas Mut2‐SETD2 had no significant effect (Figure 6B). Furthermore, RT‐PCR results showed that hnRNPL attenuated IL‐18 exon retention induced by Mut‐SETD2. These results indicate that SETD2 recruits hnRNPL to the IL‐18 pre‐mRNA through the SHI domain to promote IL‐18 exon retention. Furthermore, we explored the relationship between PTBP3‐mediated exon skipping and hnRNPL‐induced exon retention of IL‐18. Knockdown of hnRNPL enhanced the effect of PTBP3 on IL‐18 exon skipping, whereas knockdown of PTBP3 did not significantly enhance hnRNPL‐induced IL‐18 exon retention (Figure 6D,E). These results suggested that hnRNPL interferes with PTBP3‐mediated IL‐18 exon skipping, which in turn promotes IL‐18 exon retention. Because hnRNPL and PTBP3 bind in close proximity to the IL‐18 pre‐mRNA, we speculated whether the two proteins interact with each other (Figure S5G, Supporting Information). Immunoprecipitation results confirmed that PTBP3 could bind to hnRNPL, even in the presence of RNase A, indicating direct binding of the two proteins (Figure 6F). Based on the domain characteristics of PTBP3 and hnRNPL, we constructed various deletion mutants (Figure S5H and I, Supporting Information).^[^ ^43^, ^44^ ^]^ We found that hnRNPL was unable to bind to the PTBP3 mutants lacking the RRM3 domain, whereas PTBP3 was unable to bind to the hnRNPL mutants lacking the RRM2 domain (Figure 6G,H). The results of molecular docking also suggested strong binding between PTBP3 and hnRNPL (Figure 6I). Further, there was no significant change in alternative splicing of IL‐18 after transfection of hnRNPL‐Δ2 in NOZ, suggesting that hnRNPL interferes with PTBP3‐mediated exon skipping of IL‐18 by binding to PTBP3 (Figure 6J). We found that the ability of MRG15 to bind to PTBP3 was not altered after hnRNPL overexpression (Figure S5J, Supporting Information). However, hnRNPL overexpression attenuated the binding of PTBP3 to IL‐18 pre‐mRNA, and hnRNPL knockdown enhanced the binding of PTBP3 to IL‐18 pre‐mRNA (Figure 6K,L). Taken together, these results show that SETD2/hnRNPL affect the exon‐skipping function of PTBP3 by interfering with its ability to bind to IL‐18 pre‐mRNA.

## 3 Discussion

It is well recognized that alternative splicing abnormalities can produce aberrant proteins that lead to various aspects of tumorigenesis, including proliferation, invasion, metastasis, angiogenesis, and immune escape.^[^ ^45^ ^]^ Trans‐acting splicing factors are a class of alternative splicing‐associated RBPs that recognize and bind to cis‐regulatory elements on pre‐mRNA, facilitating or inhibiting the inclusion of the exon into the mature mRNA.^[^ ^46^ ^]^ Dysregulation of these splicing factors, which are usually RBPs, often leads to multiple aberrant alternative splicing events in tumors. Consistent with our findings in patients with gallbladder cancer, PTBP3 is highly expressed in multiple tumors and is associated with poor prognosis.^[^ ^14^, ^16^, ^47^, ^48^ ^]^ PTBP3 can maintain UBE4A mRNA stability to promote colorectal cancer cell proliferation^[^ ^14^ ^]^ and can also promote gastric cancer metastasis by mediating alternative splicing in CAV1.^[^ ^15^ ^]^ Interestingly, we found that knockdown of PTBP3 did not significantly alter the biological behavior of gallbladder cancer cells. Recent pan‐cancer analyses have demonstrated that PTBPs are prognostic biomarkers associated with immunomodulation.^[^ ^49^ ^]^ It has also been shown that PTBP2 has no significant effect on neuroblastoma cell viability, whereas PTBP2‐mediated alternative splicing of IRF9 facilitates immune compartmentalization in neuroblastoma.^[^ ^50^ ^]^ Therefore, it is reasonable to hypothesize that PTBP3 contributes to gallbladder cancer progression by affecting the tumor microenvironment. Despite the high degree of malignancy in gallbladder cancer, little is known about the immunosuppressive tumor microenvironment.

Based on bioinformatics analysis and cell biology experimental validation, we demonstrated that PTBP3 was able to promote exon skipping of IL‐18, which generated ΔIL‐18, an isoform expressed only in tumor tissues. IL‐18, a myeloid leukocyte inflammatory mediator, induces IFNγ secretion from T cells and NK cells, and has been shown to confer anti‐tumor activity.^[^ ^33^ ^]^ One study found an mRNA isoform of another important interleukin, IL‐15, that was preferentially expressed in certain human tumors. The tumor isoform encoded an IL‐15 protein with a 21‐amino‐acid signal peptide (SP), which was shorter than the classic IL‐15 SP (48 amino acids).^[^ ^51^, ^52^ ^]^ Only 48SP‐IL‐15 enhanced protective responses against pathogens, whereas 21SP‐IL‐15 appeared to be immunosuppressive.^[^ ^53^ ^]^ Thus, combined with sequencing data and clinical sample validation, we can speculate that alterations in splicing during gallbladder cancer progression may favor the production of ΔIL‐18, the isoform involved in immune escape. In recent years, immunotherapy represented by PD‐1/PD‐L1 immune checkpoint inhibitors is gradually changing the landscape of tumor treatment. However, there are still a large number of patients who cannot benefit from this kind of immunotherapy, and expanding the beneficiary population is an ongoing challenge.^[^ ^54^ ^]^ In order to resist immunotherapy, tumors may employ multiple “immune escape” strategies. Tumor immune escape, one of the features necessary for tumor development, is a complex mechanism involving the participation of genes, metabolism, inflammation, vasculature, and various other components.^[^ ^55^ ^]^ There have been few studies on immune escape in gallbladder cancer. In one study, genomic ERBB2/ERBB3 mutations were shown to promote PD‐L1‐mediated immune escape in gallbladder cancer.^[^ ^56^ ^]^ Recent studies have also shown that increased PD‐1 expression is associated with poor prognosis and immune microenvironment heterogeneity in gallbladder cancer.^[^ ^57^ ^]^

We found that PTBP3 promotes immune escape from gallbladder cancer through ΔIL‐18, both in vitro and in vivo. Mechanistically, we discovered that ΔIL‐18 reduced FBXO38‐mediated PD‐1 ubiquitination by downregulating FBXO38 transcription. Immune escape is significantly influenced by PD‐1/PD‐L1, which controls the maintenance and induction of immune tolerance and escape from the tumor microenvironment.^[^ ^58^ ^]^ RNA‐based approaches such as antisense oligonucleotides (ASO), which target exon skipping by combining specific sequences, are a promising therapeutic approach.^[^ ^59^ ^]^ Several ASO drugs that target exon skipping have been approved by the FDA for the treatment of Duchenne muscular dystrophy.^[^ ^60^ ^]^ We also designed and synthesized an ASO targeting IL‐18 exon skipping, which has excellent anti‐tumor effects both in vivo and in vitro, with a good safety profile. Enhancing the anti‐tumor effect of CD8+T cells by blocking the production of ΔIL‐18 with an ASO provides a new perspective for gallbladder cancer immunotherapy. In addition, our study also showed that the pro‐tumorigenic effects of the PTBP3/ΔIL‐18 axis were CD8+ T cell‐dependent or immune‐independent. Some research results show that microsatellite instability (MSI) and mismatch repair‐deficiency (dMMR) is closely related to the occurrence and development of gallbladder cancer.^[^ ^61^ ^]^ MSI and dMMR is one of the hotspots of molecular biology research in recent years, and the results of some studies have shown that the microsatellite‐instability‐high tumor immunotherapy effect and prognosis are better, which can be used for tumor survival assessment.^[^ ^62^ ^]^ Our study is relatively limited in this piece due to the limitations of in vivo modeling as well as limited clinical samples. If the relationship between ΔIL‐18 and MSI can be demonstrated in future studies, I believe that it may be possible to improve the survival benefit for patients with gallbladder cancer. On the other hand, however, the mechanism by which ΔIL‐18 affects FBXO38 transcript levels in CD8+ T cells require further investigation. In addition, ΔIL‐18 protein lacks only 4 amino acids compared to cIL‐18 protein, therefore current antibodies and ELISA reagents for IL‐18 protein cannot distinguish between cIL‐18 and ΔIL‐18. Due to the specificity of ΔIL‐18 expression in tumor tissue, the design of early detection kits for ΔIL‐18 holds great promise and may help to improve the detection rate of gallbladder cancer.

Chromatin is a highly dynamic structure that plays a crucial role in transcriptional and co‐transcriptional regulation.^[^ ^63^ ^]^ A growing body of research suggests that alternative splicing occurs in a co‐transcriptional manner, and experimental and computational evidence also suggests that chromatin dynamics can influence the splicing process.^[^ ^64^, ^65^, ^66^ ^]^ Posttranslational histone modifications are the most widely studied epigenetic phenomena, and are associated with various biological processes, including RNA splicing.^[^ ^67^ ^]^ It was shown that these modifications can regulate alternative splicing by recruiting splicing factors.^[^ ^38^, ^67^ ^]^ Studies investigating the relationship between splicing and histone modifications have focused on H3K36me3 in particular. H3K36me3 binds to PSIP1/p52, which, in turn, recruits the splicing factor SRSF1, promoting alternative splicing.^[^ ^68^ ^]^ It has also been shown that EAF3, which binds to H3K36me3, can interact with the splicing factor PRP45 during splicing activation.^[^ ^69^ ^]^ In this study, we reveal that H3K36me3 is able to bind to the histone tail‐binding protein MRG15, which in turn recruits PTBP3 to regulate exon skipping of IL‐18. Moreover, PTBP3‐mediated exon skipping of IL‐18 is histone modification‐dependent. This study innovatively identified the two‐sided nature of the H3K36 methyltransferase SETD2 in regulating IL‐18 alternative splicing; on the one hand, SETD2 catalyzes H3K36 methylation via the SET domain, thus recruiting MRG15/PTBP3 to promote IL‐18 exon skipping, but on the other hand SETD2 directly binds to hnRNPL via the SHI domain to interfere with PTBP3 binding to IL‐18, thereby impairing IL‐18 exon skipping.

In conclusion, we found that expression of the splicing factor PTBP3 was significantly upregulated in gallbladder cancer and correlated with the prognosis of patients with gallbladder cancer. PTBP3 did not have a significant effect on the biological behavior of gallbladder cancer cells but facilitated immune escape of tumor cells by promoting exon skipping of IL‐18. Mechanistically, ΔIL‐18 produced via exon skipping reduced PD‐1 ubiquitin‐mediated degradation in CD8+T cells by decreasing FBXO38 transcription levels. During alternative splicing regulation, H3K36me3 promotes exon skipping by recruiting PTBP3 via MRG15, and couples IL‐18 transcription and alternative splicing. Meanwhile, hnRNPL, which binds to SETD2 via the SHI domain, impairs IL‐18 exon skipping by inhibiting PTBP3 binding to IL‐18 pre‐mRNA. Our study provides a deeper understanding of aberrant alternative splicing events in gallbladder cancer progression, and provides new therapeutic targets for gallbladder cancer immunotherapy.

## 4 Experimental Section

### Patient data

All the specimens and prognostic information of the patients involved in the study were provided to the Department of General Surgery, Xinhua Hospital, Shanghai Jiaotong University School of Medicine, and the pathology specimens were confirmed by pathologists. Inclusion and exclusion criteria for patients involved: all pathologically diagnosed and not receiving adjuvant therapies such as chemotherapy, targeted therapies. All patients signed an informed consent form.

### Immunohistochemistry (IHC) and Multiplex Immunohistochemistry (mIHC)

For immunohistochemistry, tissue slides were rehydrated and incubated in 3% H_2_O_2_ for 5 min, boiled with 0.01 M sodium citrate buffer and subjected to antigen recovery, followed by blocking with 5% goat serum, and subsequently incubated with primary antibody at 4 °C overnight. The next day, slides were incubated with goat‐anti‐rabbit secondary antibody conjugated to horseradish peroxidase for 1 h at room temperature. Subsequently, the sections were stained with 3,3′‐diaminobenzidine and counterstained with hematoxylin. For multiple immunohistochemistry, tissue slides were incubated with tyramine signal‐ amplification conjugated fluorophores for 10 min at room temperature after immunohistochemical primary and secondary antibody staining. Then, all conjugates other than fluorophores were removed from the tissue slides by an antigen recovery procedure, followed by primary antibody‐secondary antibody‐TSA‐conjugated fluorophore staining (Beyotime). Finally, the slides were stained with DAPI and mounted. Three researchers independently assessed immunostaining scores. Information on all antibodies used was listed in Table S1 (Supporting Information).

### Cell Culture and Reagents

HEK293T and human gallbladder cancer cell lines (NOZ, ZJU‐0430, GBC‐SD, EH‐GB1, OCUG‐1, SGC‐996) were purchased from the cell bank of Shanghai Institutes for Biological Sciences, Chinese Academy of Sciences. All cells were cultured in DMEM high glucose medium (BasalMedia, Shanghai) containing 10% fetal bovine serum (EpiZyme, Shanghai), and placed in a cell culture incubator containing 5% concentration of CO_2_ at 37 °C. Chloroquine∖MG‐132∖PD‐1 mAb Nivolumab∖human IL‐18∖Cycloheximide used in the study were purchased from MedChemExpress.

### Cell Transfection

The siRNAs were synthesized by Genepharma company (Shanghai, China). siRNA sequences were shown in Table S2 (Supporting Information). siRNAs were transfected using Rfect (Baidai, China) reagent according to the instructions. Full‐length cDNAs of the genes involved in the study were cloned and produced by Longqian Biotechnology (Shanghai, China). The plasmids were transfected with Lipofectamine 2000 according to the transfection instructions. ASOs with 2′‐Omethoxyethy modification targeting IL‐18 exon skipping were synthesized and purified by HuageneBIO (Shanghai, China). Control group ASO sequences (UCACUUCCUCCUCCCUCCCC) do not target any other targets.

### Cell Proliferation Assay

For Cell Counting Kit‐8 (Yeasen, China), the procedure was performed according to the instructions in order to detect cell proliferation capacity. For EdU‐488 DNA synthesis assay (Beyotime, China), briefly, treated cells were incubated with EDU for 2 h, then fixed, washed. Next, Click Additive Solution was added and incubated for 30 min, finally stained with DAPI.

### RNA Fluorescent In Situ Hybridization (FISH) and Immunofluorescence (IF)

For FISH, cells were fixed with 4% paraformaldehyde for 15 min, followed by permeabilization for 30 min, and finally the cells were incubated overnight with diluted IL‐18 probe (5′‐TCCCCCTCTTAGCTGAAGATGATGGGTAAA‐3′) in RNA FISH hybridization buffer (1:50). For IF, cells were first fixed with 4% paraformaldehyde for 15 min, then cells were washed and blocked, then incubated with diluted specific primary antibody at 4 °C overnight.

### Reverse Transcription PCR (RT‐PCR) and Quantitative Real‐Time PCR (qPCR)

RNA from clinical sample tissues and cells was extracted by Trizol (Sangon, Shanghai) according to the instructions. Extracted RNA was reverse transcribed using HiScript II 1st Strand cDNA Synthesis Kit (Vazyme, China). HiScript II One Step RT‐PCR Kit (Vazyme, China) was used in RT‐PCR ChamQ SYBR qPCR Master Mix (Vazyme, China) was used for qPCR according to the instructions and primers used for amplification were listed in Table S3 (Supporting Information).

### Protein Expression and Purification

The cDNA of ΔIL18 mutant (residues 22–25 deletion) was synthesis (Longqian Biotechnology) and cloned into pET28‐SMT3a vector between BamHI and SalI sites, which carried a 6×His–SUMO tag at the N terminus. Recombinant 6×His–SUMO‐ΔIL18 protein was expressed in E. coli strain BL21(DE3) Gold (Agilent). Cultures were grown in LB media at 37 °C to OD600 of 0.6. After induction with 0.2 mM IPTG, the cells were grown at 18 °C overnight. The cells were harvested by centrifugation at 4 °C and disrupted by French Press (JNBio) in buffer 1 (20 mM Tris‐HCl, pH 8.0, 25 mM Imidazole, 500 mM NaCl, and 1 mM PMSF). The bacterial lysate was clarified by centrifugation at 18 000 rpm for 1 h. The supernatant of recombinant 6×His–SUMO‐ΔIL18 protein was loaded onto HisTrap HP column (Cytiva). After extensive washing with buffer 1, 6×His–SUMO‐ΔIL18 was eluted by buffer with a linear imidazole gradient from 25 to 500 mM. Ulp was added to cleavage the 6×His–SUMO tag. ΔIL18 was dialysis into buffer S (20 mM Tris‐HCl, pH 8.0, and 500 mM NaCl), and flow through the HisTrap HP column (Cytiva) for second time to remove the 6×His–SUMO tag. Then ΔIL18 were further purified by size‐exclusive chromatography using a Superdex G200 HiLoad 16/60 column (Cytiva) in PBS buffer (HyClone). The peak fraction was pooled and concentrated into 20 mg mL^−1^ for the further experiment.

### CD8+T Cell Extraction and Culture

CD8+T cells were extracted from peripheral blood of healthy individuals using EasySep Direct Human CD8+T Cell Isolation Kit (Catalog #19 663, Stemcell) according to the instructions. Extracted CD8T cells were cultured in ImmunoCult‐XF T Cell Expansion Medium (Catalog #10 981, Stemcell) and placed in a cell culture incubator containing 5% concentration of CO_2_ at 37 °C. To activate CD8+T cells, the medium was supplemented with 5 ng mL^−1^ of Human Recombinant IL‐2 (Catalog #78 036, Stemcell) and 25 µL mL^−1^ of ImmunoCult Human CD3/CD28/CD2 T Cell Activator (Catalog #10 970, Stemcell).

### Western Blotting

First, we selected different SDS‐PAGE gels (7.5%∖10%∖15%) (EpiZyme, Shanghai) concentrations depending on the molecular weight of the protein. Protein samples were transferred to PVDF films (Millipore) after constant pressure electrophoresis. And after the films were blocked by Protein Free Rapid Blocking Buffer 1× (EpiZyme, Shanghai), it was incubated overnight at 4 °C with the corresponding diluted primary antibody. The following day, after washing by TBST (Sangon, Shanghai), the films were blocked with HRP‐labeled secondary antibody (Beyotime) for 1 h, finally detected with an enhanced chemiluminescence kit (Vazyme, China). Information of the antibodies involved was listed in Table S1 (Supporting Information).

### Olink Proteomics

Cell supernatant proteins were measured using an Olink Immuno‐Oncology panel (Olink Proteomics AB, Uppsala, Sweden) according to the manufacturer's instructions. Briefly, pairs of oligonucleotide‐labeled antibody probes bind to the target protein. The addition of DNA polymerase results in a DNA polymerization reaction that produces a unique PCR target sequence. Subsequently using a microfluidic real‐time PCR instrument (Signature Q100, LC‐Bio Technology Co.) The data were then quality controlled and normalized using an internal extension control and an inter‐plate control to adjust for intra‐ and inter‐run variation.

### RNA Immunoprecipitation (RIP) and RIP Sequencing (RIP‐seq)

RNA Immunoprecipitation Kit (BersinBio, China) was used for RIP in the study and was performed according to the instructions. RNA libraries were constructed using the ASMARTer Universal Low‐Input RNA Sequencing Kit and sequenced on an illumina NovaSeq 6000 from LC Biotech (Hangzhou, China). The antibodies and primers involved were listed in Table S1 (Supporting Information).

### mRNA Sequencing (mRNA‐seq)

RNA from clinical sample tissues and cells was extracted by Trizol (Invitrogen) according to the instructions. The RNA libraries were constructed on the illumina NovaseqTM 6000 platform by LC Bio Technology CO.,Ltd (Hangzhou, China). Bioinformatics analysis involved in the article on OmicStudio (https://www.omicstudio.cn/tool).

### Immunoprecipitation (IP)

Protein lysates from treated cells were added to protein A/G magnetic beads (MedChemExpress) conjugated with the corresponding antibodies and incubated overnight at 4 °C with rotation. Then the magnetic beads were washed, and the proteins were extracted by boiling with the addition of 1×Loading Buffer (Beyotime). Finally, western blotting was used for subsequent experiments.

### Flow Cytometry

Apoptosis and HuPBMC reconstitution efficiency were validated using flow cytometry. For apoptosis detection, Annexin V‐FITC kits (Yeasen, China) were used as provided by the manufacturer. The proportion of human CD45‐positive PBMC in peripheral blood samples from mice was determined using PE‐CD45 antibody (Biolegend, USA). And samples were analyzed using a flow cytometer (CytoFLEX, Beckman Coulter).

### ELISA

Cells were treated accordingly, and supernatant medium was collected to detect the levels of IL18, Granzyme B and IFNγ using Human IL‐18 ELISA Kit (Abcam, ab215539), Human Granzyme B ELISA Kit (ab235635) and IFNγ ELISA Kit (Abcam, ab174443).

### RNA Pulldown

It was synthesized biotin‐labeled probes for the corresponding genes and performed RNA pull‐down analyses following standard procedures. Briefly, RNA samples were incubated with protein extracts from NOZ cells and streptavidin Dynabeads (Vazyme, China). Subsequent experiments were then analyzed by western blotting.

### Chromatin Immunoprecipitation (CHIP)

CHIP experiments using SimpleChIP Enzymatic Chromatin IP Kit (Cell Signaling Technology) operating according to the instructions was performed. Enriched DNA fragments were subsequently confirmed using DNA electrophoresis or by qPCR. The antibodies and primers involved were listed in Tables S1 and S3 (Supporting Information), respectively.

### Dual Luciferase Assay

The sequence containing the FBXO38 promoter was cloned and produced by Longqian Biotechnology (Shanghai, China). Cells were treated accordingly, and luciferase activity was determined using a Dual‐Luciferase Reporter Assay (Yeasen, China) according to the instructions.

### Hematoxylin and Eosin Staining (HE)

HE staining's of mouse tissues were performed using Hematoxylin and Eosin Staining Kit (Beyotime) according to the instructions.

### Animal

NOG mice (female) purchased from the Shanghai Laboratory Animal Center of the Chinese Academy of Sciences (Shanghai, China). The mice used were randomized into different groups. No mice died accidentally during the experiment. HuPBMC modeling was conducted with reference to what has been reported in the literature.^[^ ^18^ ^]^ Peripheral blood PBMC from healthy adult donors were injected into 4‐week‐old NOG mice via the tail vein, and one week later, 2 × 10^6^ treated NOZ cells were inoculated into the axilla of mice. The subsequent experimental procedure has been described exhaustively in the figures in the article. One month later, all HuPBMC mice were euthanized, and tumor specimens were removed for subsequent analysis. Animal experiments were approved by the Ethics Committee of Xinhua Hospital (Animal experiment approval number: XHEC‐NSFC‐2021‐009, approval date: February 18, 2021).

### Docking and Visualization of Protein Simulations

We used the PDB database to download the 3D structure diagram of PTBP3 (O95758) and HNRPL (P14866), saving in PDB format. Pymol2.3.0 was used to remove protein crystal water and original ligands, and the protein structure was imported into AutoDocktools (v1.5.6) for hydrogenation, charge calculation, charge assignment, atom type assignment, and saved in “pdbqt” format, then imported them into AutoDock software for molecular docking. The search space: size_x: 50, size_y: 50, size_z: 50 each lattice spacing is 0.375 (A) and exhaustiveness: 10, the rest of the parameters as the default Settings. Docking results were visualized using PyMol software.

### Statistical Analysis

PRISM 8.0.1GraphPad was used to perform the statistical analysis and do the statistical related plots. Student's t‐test was used for statistical analysis between the two groups involved in the study. In the study we defined P‐value less than 0.05 as statistically significant (P<0.05: *; P<0.01: **; P<0.001: ***). All statistical analyses were based on three independent replication experiments. Univariate survival analysis was also performed using the Kaplan‐Meier test.

## Conflict of Interest

The authors declare no conflict of interest.

## Ethics Approval and Consent to Participate

Animal experiments involving mice met the requirements and were approved by Xinhua Hospital of Shanghai Jiao Tong University School of Medicine (Animal experiment approval number: XHEC‐NSFC‐2021‐009, approval date: February 18, 2021).

## Supporting information

Supporting Information

## Data Availability

The data that support the findings of this study are available from the corresponding author upon reasonable request.
