# Supplementary material for: PTBP3 Mediates IL‐18 Exon Skipping to Promote Immune Escape in Gallbladder Cancer
Source: Adv Sci (Weinh). 2024 Aug 8;11(38):2406633. doi: 10.1002/advs.202406633 (PMC11481411; doi:10.1002/advs.202406633)
Supplement: Supplementary file 1 — Supporting Information [file ADVS-11-2406633-s001.docx]

Supplementary Table1. Antibody information

| Antibody | Application | Company | Catalogue |
| --- | --- | --- | --- |
| PTBP3 | IHC/mIHC/Western blotting/IP | Thermo Fisher Scientific | PA5-98125 |
| CD8A | mIHC | Cell Signaling Technology | 85336 |
| CK19 | mIHC | Abcam | ab223259 |
| CD3 | mIHC | Abcam | ab11089 |
| Myc | Western blotting | Abcam | ab32 |
| Flag | Western blotting | Abcam | ab205606 |
| SETD2 | Western blotting | Cell Signaling Technology | 23486 |
| Histone H3 | Western blotting/CHIP | Abcam | ab1791 |
| H3K36me3 | Western blotting/CHIP | Abcam | ab9050 |
| H3K4me3 | Western blotting/CHIP | Abcam | ab8580 |
| MRG15 | Western blotting/IP | Cell Signaling Technology | 14098 |
| PD-1 | Western blotting | Abcam | ab52587 |
| PD-L1 | Western blotting | Abcam | ab205921 |
| UB | Western blotting | Cell Signaling Technology | 58395 |
| FBXO38 | Western blotting/IP | Abcam | ab87729 |
| hnRNPL | Western blotting/IP | Abcam | ab6106 |
| GAPDH | Western blotting | Abcam | ab8245 |

Supplementary Table2. siRNA sequence

| Target | siRNA sequence |
| --- | --- |
| Negative Control | TTCTCCGAACGTGTCACGT |
| PTBP3 si-1 | GAGUGAAGAUUAUGUUUAATT |
| PTBP3 si-2 | CCCAGUAAAUGCACAUUAUTT |
| PTBP3 si-3 | GCCCUGUGCUUCGAAUAAUTT |
| FBXO38 si | GACTTCCTTTGTATCAGCTTA |
| MRG15 si-1 | AAGCAGAAACAGCGAGAACTT |
| MRG15 si-2 | AAGCCAAUCAGGAGCAGUATT |
| hnRNPL si-1 | GCAGCCGACAACCAAAUAUTT |
| hnRNPL si-2 | GCUUGGAUCAAUCUAAGAUTT |
| SETD2 si-1 | GAAACCGUCUCCAGUCUGUTT |
| SETD2 si-2 | GAGAGGUACUCGAUCAUAA |
| ASH2 si-1 | CCGAGUAACUAACUUAUUUAA |
| ASH2 si-2 | CCCGUUUAACAAAGAUGGCUA |

Supplementary Table3. Primer sequence

| Target | Primer sequence |
| --- | --- |
| PTBP3 | F:TTCTCCGAACGTGTCACGT |
|  | R:GAGUGAAGAUUAUGUUUAATT |
| MRG15 | F: GGAGGCGGCGAATCACTTAT |
|  | R: CCCATTCATCCCAACCTCCT |
| ASH2 | F: GGGAGTGCATGACAACCAGA |
|  | R: TCCCACTAGTAGACACAGCA |
| hnRNPL | F: TACGCAGCCGACAACCAAATA |
|  | R: CTCCGGGAGTCATCCGAGT |
| SETD2 | F: CCCGACCCCTGAGCAAAG |
|  | R: GCCAAGTGCAGTGAGAAACC |
| IL-18 2-4 | F: GCATCAACTTTGTGGCAATG |
|  | R: CAAAGTAATCTGATTCCAGG |
| IL-18 Exon1 | F: GTCTCCCAGTGCATTTTGCC |
|  | R: TTGTTGCGAGAGGAAGCGAT |
| IL-18 Intron1 | F: TCACTACGATGAGCAGTCAGT |
|  | R: AGTGTAAAGGGAACATGAGAGCA |
| IL-18 Intron2 | F: GCTTCATTTTATGGGCCTTTTGG |
|  | R: GCTACTCCTCTTTCTCGAACTCC |
| IL-18 Exon2 | F: TGTTTCTCTCTTTTTCCCCCTCT |
|  | R:TGAGATAGATGTGTCTCAAGCAGA |
| IL-18 Exon3 | F: TCCCCCTCTTAGCTGAAGATGA |
|  | R: TCCTCAGCTGACAATGGTGA |
| IL-18 Intron3 | F: TGTGGACTCAGTAGCACAGC |
|  | R: AGGACCTATGAAACAGGCAAAGT |
| IL-18 Exon4 | F: AACCTGGAATCAGATTACTTTGGC |
|  | R: AGAGGCCGATTTCCTTGGTC |
| IL-18 Intron4 | F: GGAAACTTTATAAGGCATCCACGT |
|  | R: CCACACCTGGTAAACTATCAGC |
| IL-18 Exon5 | F: ATGCACCCCGGACCATATTT |
|  | R: TCTCACAGGAGAGAGTTGAAATT |
| IL-18 Intron5 | F:CAGAAATAACAAGAAGCAGAGAACCA |
|  | R: TGTTCAGGAGATGCAGGTCA |
| IL-18 Exon6 | F: TCAGAGAAGTGTCCCAGGAC |
|  | R: TCTATCCCCCAATTCATCCTCT |
| IL-18 Exon3-4 | F: TGACCAAGGAAATCGGCCTC |
|  | R: CCATACCTCTAGGCTGGCTA |
| FBXO38 | F: GGGTCGGCGTAGGTACTTTG |
|  | R: CTTTCGTGGCCCCATTGTTG |
| PD-1 | F: AGATCAAAGAGAGCCTGCGG |
|  | R: CTCCTATTGTCCCTCGTGCG |
| GAPDH | F: CAACAGCCTCAAGATCATCAGC |
|  | R: TTCTAGACGGCAGGTCAGGTC |

Supplementary Table4. RNA binding proteins

| SRSF12 | PTBP1 | BUB3 | POLR2B | RBM15B | G3BP2 |
| --- | --- | --- | --- | --- | --- |
| ELAVL2 | SF3A2 | CWC27 | NONO | LUC7L3 | GOLGB1 |
| MSI1 | SRRT | ESS2 | CDC5L | DDX39B | HNRNPCL1 |
| CELF4 | RBMXL1 | MBNL3 | RBMX2 | NSRP1 | HNRNPDL |
| YBX1 | RBM14 | GPATCH1 | SNRNP40 | RBM23 | LSM11 |
| SRPK1 | ESRP2 | SNRNP27 | WBP11 | FUS | MARK2 |
| SNRPG | SNRNP48 | SRSF10 | PRPF38A | YJU2 | METAP2 |
| PABPC1 | DHX38 | SMU1 | NCBP1 | CRNKL1 | NAA15 |
| KHDRBS3 | RBM4 | PPIL4 | SF3B4 | SUGP1 | NOLC1 |
| MAGOH | DHX15 | CHERP | SMNDC1 | RBM22 | PARN |
| SNRPF | DHX16 | SNRNP200 | NCBP2 | SNRNP35 | PKM |
| RBM38 | PRPF8 | CCAR1 | KIN | MBNL1 | PRRC2C |
| DDX39A | DDX23 | STAU1 | SRSF3 | CELF5 | PSPC1 |
| LSM2 | PCBP2 | HNRNPU | RBM28 | CSTF2T | PUS1 |
| SNRPA1 | RBM10 | RBM42 | ISY1 | SF3B1 | RBM34 |
| PPIL1 | ZNF207 | DDX41 | U2AF1 | DHX8 | RBM45 |
| ILF2 | CDC40 | FUBP1 | NUDT21 | CLK2 | SERBP1 |
| SNRPC | PRPF6 | TCERG1 | HNRNPA3 | HNRNPA0 | SLTM |
| SF3B6 | ZMAT2 | HNRNPK | SRSF1 | HNRNPH1 | SSRP1 |
| SNRPE | NOSIP | CPSF1 | HNRNPA1L2 | TRA2A | SUCLG1 |
| C1QBP | PTBP2 | U2AF2 | EFTUD2 | DHX35 | SUPV3L1 |
| PSIP1 | SRRM1 | GEMIN5 | HNRNPA2B1 | DDX5 | TAF15 |
| CPSF3 | ADAR | EWSR1 | PRPF3 | RBM19 | TBRG4 |
| ESRP1 | SNW1 | PCBP1 | SRSF9 | GTF2F1 | TUFM |
| PCBP3 | NAA38 | HNRNPH3 | SRSF2 | RBM41 | ZNF106 |
| TXNL4A | PPIG | RBM6 | CSTF2 | MYEF2 | ZNF622 |
| SF3B3 | WDR33 | LUC7L | PRMT5 | XAB2 | SAFB |
| SNRPB2 | GPKOW | SNRNP70 | BCAS2 | YTHDC1 | APOBEC3C |
| PUF60 | RBM7 | RBM47 | HNRNPA1 | SCAF8 | DDX3Y |
| PPIH | TIAL1 | RNPC3 | SND1 | CASC3 | SAFB2 |
| LSM4 | ZMAT5 | DDX17 | DHX57 | PPIL2 | ZC3H8 |
| HNRNPAB | LUC7L2 | SREK1 | RNPS1 | FASTK | DBR1 |
| PHF5A | SRSF4 | RBM11 | PRCC | MTREX | RAVER2 |
| SNRPD2 | CELF1 | SRRM2 | TRA2B | DDX3X | AKAP17A |
| RBM17 | TFIP11 | CLK4 | PTBP3 | LGALS3 | CELF2 |
| UPF3B | ZNF326 | SRSF5 | KHSRP | SLU7 | HNRNPH2 |
| SNRPA | SNIP1 | CLK1 | RBM15 | AQR | SNRPN |
| SCNM1 | DDX46 | RBM5 | KHDRBS1 | CD2BP2 | RBFOX1 |
| SF3A3 | SF3A1 | RBPMS | PLRG1 | PRPF40B | ACO1 |
| SNRPD3 | SART3 | NOVA2 | SRSF7 | AKAP8L | AKAP8 |
| SNRPB | SRPK3 | TTC14 | CSTF1 | RBPMS2 | BOP1 |
| DNAJC6 | PNN | RBM20 | HNRNPL | SRSF11 | CCDC86 |
| SYNCRIP | CCDC12 | CIRBP | CPSF4 | CCAR2 | CEBPZ |
| SF3B5 | WBP4 | NCBP2L | PPP1R8 | RBM39 | DAZ3 |
| LSM6 | RBM4B | RBM24 | PRPF4 | AGGF1 | DDX19B |
| USP39 | ZCCHC8 | KHDRBS2 | GCFC2 | ZRSR2 | DDX47 |
| BUD31 | LSM8 | CELF6 | HNRNPC | SFSWAP | EEF2 |
| HTATSF1 | CPSF7 | NOVA1 | HNRNPLL | SCAF11 | ESF1 |
| EIF4A3 | PRPF38B | RBFOX3 | ZC3H18 | ZC3H10 | ETF1 |
| LSM5 | UPF1 | HNRNPF | BUD13 | RBM25 | SF3B2 |
| LSM3 | ZRANB2 | CPSF2 | PPIE | SRSF6 | SMN2 |
| POLR2G | PPIL3 | CELF3 | SMN1 | RBFOX2 | ILF3 |
| DDX1 | DDX42 | HNRNPM | SNRNP25 | SUGP2 | PABPN1 |
| LSM7 | QKI | RAVER1 | U2SURP | PPWD1 | ZCRB1 |
| RBM8A | SRSF8 | SART1 | DHX9 | CACTIN | PRPF40A |
| CWC15 | IK | WTAP | FMR1 | MBNL2 | DDX20 |
| PQBP1 | PRPF4B | CDK12 | DAZAP1 | POLR2A | FRG1 |
| ELAVL1 | RBM3 | CTNNBL1 | RNF113A | MATR3 | HNRNPR |
| PRPF18 | SRPK2 | PRPF19 | TARDBP | CLK3 | TOE1 |
| HNRNPD | SF1 | FKBP4 | FIP1L1 | CCNL1 | SFPQ |
| SNU13 | SYF2 | FTO | RBMX | PRPF31 | DNAJC8 |
| RALY | TIA1 | FUBP3 | CWC22 | HNRNPUL1 |  |

Supplementary figures

Supplementary figure1


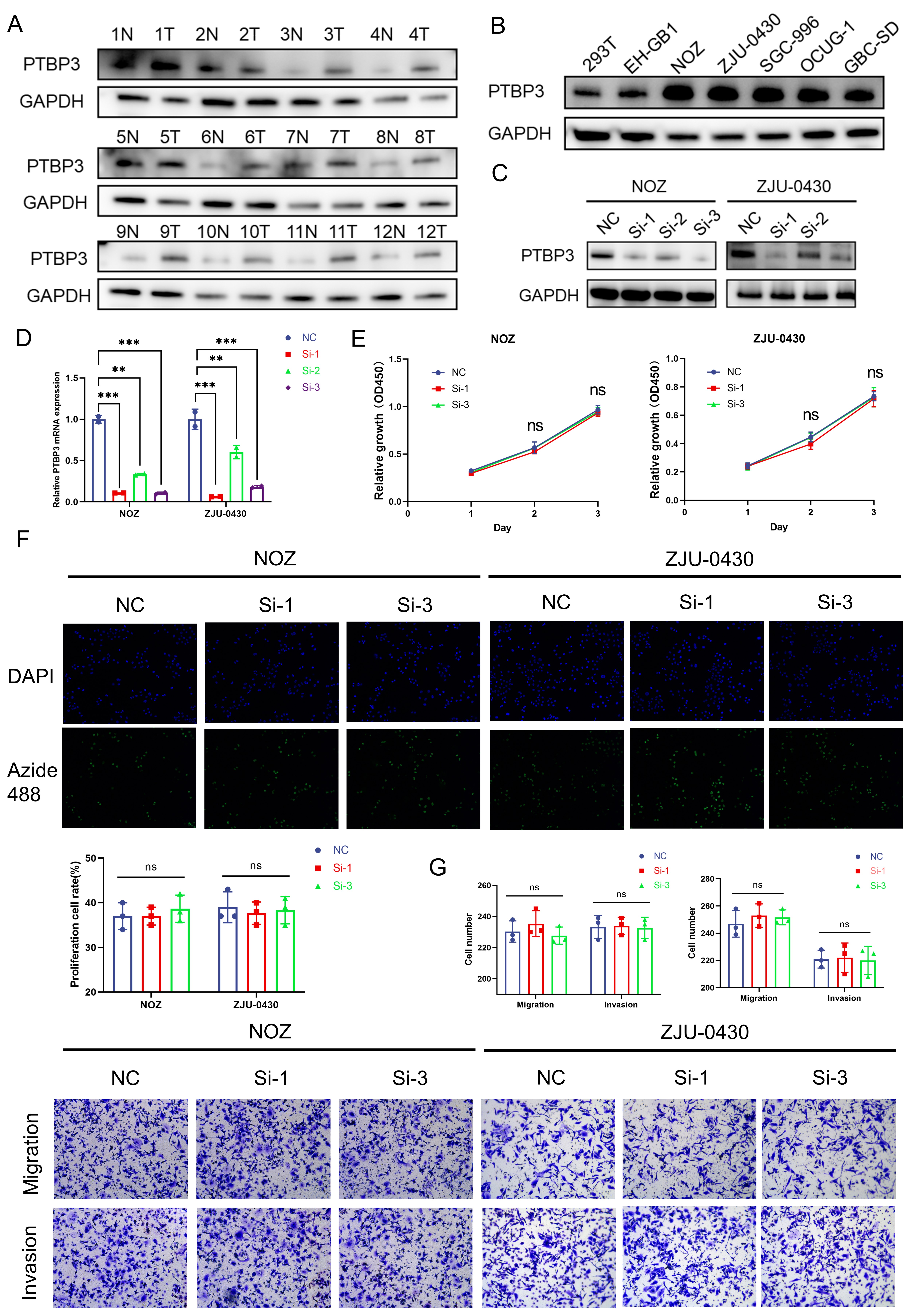


Supplementary Figure1. A. Western blotting of the PTBP3 protein levels in 12 pairs of GBC. B. Western blotting of the PTBP3 protein levels in GBC cell lines and HEK293T cell. C. Western blotting of PTBP3 siRNA knockdown efficiency validation in NOZ and ZJU-0430. D. qPCR of PTBP3 siRNA knockdown efficiency validation in NOZ and ZJU-0430. E. CCK-8 assay results of the effect of PTBP3 knockdown on GBC cell proliferation. F. EDU DNA synthesis results of the effect of PTBP3 knockdown on GBC cell proliferation. G. Transwell assays with and without Matrigels to explore the effects of PTBP3 on migration and invasion ability of GBC cells. Statistical tests involved: *P<0.05, **P<0.01, Student's t-test; Data are expressed as mean±SD, n=3.

Supplementary figure2


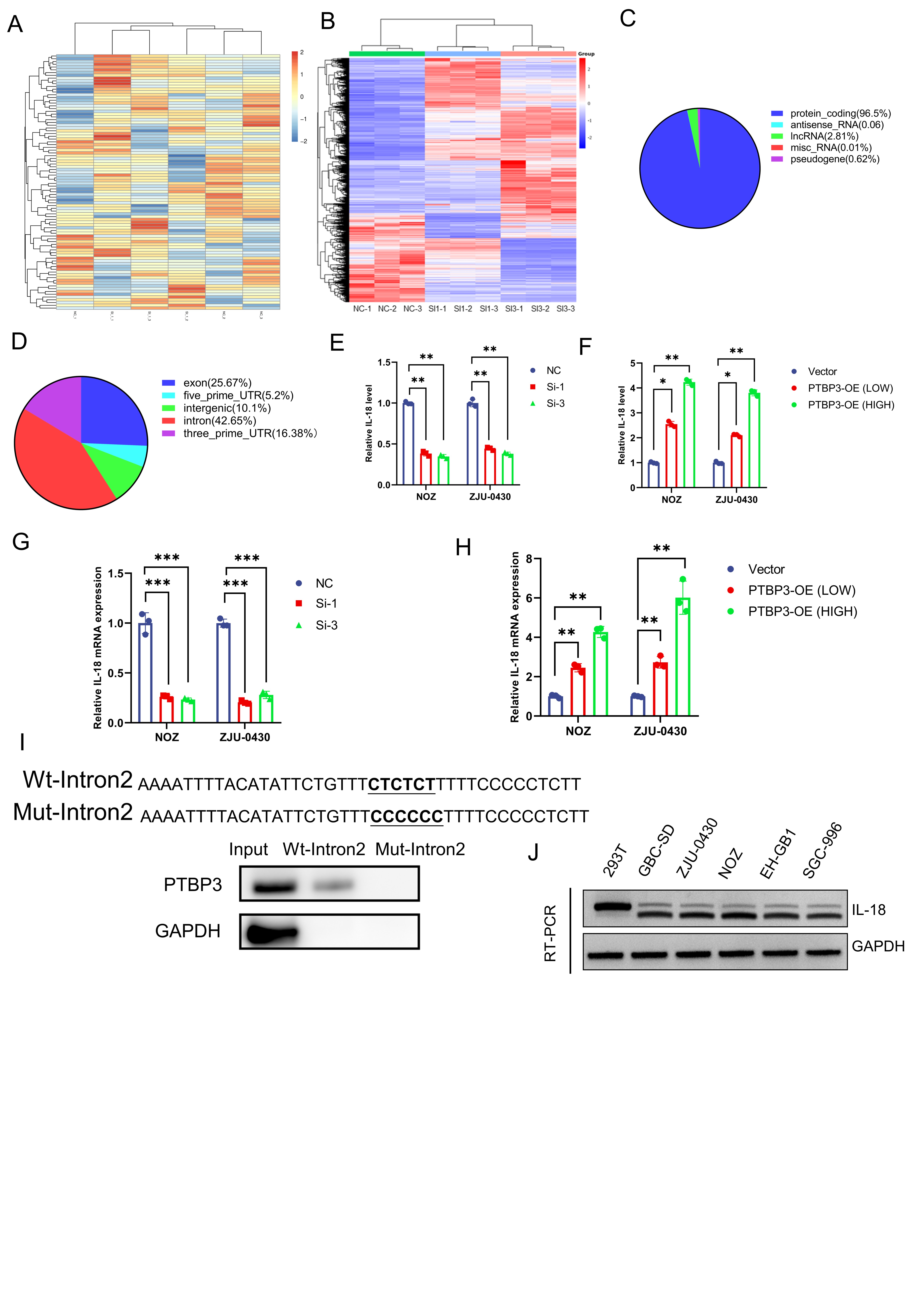


Supplementary Figure2. A. Heatmap of Differential proteins in Olink proteomics. B. Heatmap of Differential genes in mRNA-seq under PTBP3 knockdown. C. Pie chart of RNA types bound by PTBP3 in RIP-seq with anti-PTBP3 antibody. D. Pie chart of RNA positions bound by PTBP3 in RIP-seq with anti-PTBP3 antibody. E. ELISA assay to analyze changes in supernatant IL-18 after knockdown of PTBP3 in NOZ and ZJU-0430 . F. ELISA assay to analyze changes in supernatant IL-18 after overexpression of PTBP3 in NOZ and ZJU-0430. G. qPCR assay to analyze changes of IL-18 mRNA expression after knockdown of PTBP3 in NOZ and ZJU-0430. H. qPCR assay to analyze changes of IL-18 mRNA expression after overexpression of PTBP3 in NOZ and ZJU-0430. I. The PCR products of PTBP3 binding sites (Wt-Intron2\Mut-Intron2) were applied to an in vitro transcription assay with Biotin-labeled. Binding of these RNAs with PTBP3/GAPDH was detected by an RNA pull-down assay in NOZ cells. J. Detection of two isoforms of IL-18 expression in 5 gallbladder cancer cell lines and HEK293T cell line using RT-PCR assay. Statistical tests involved: *P<0.05, **P<0.01, Student's t-test; Data are expressed as mean±SD, n=3.

Supplementary figure3


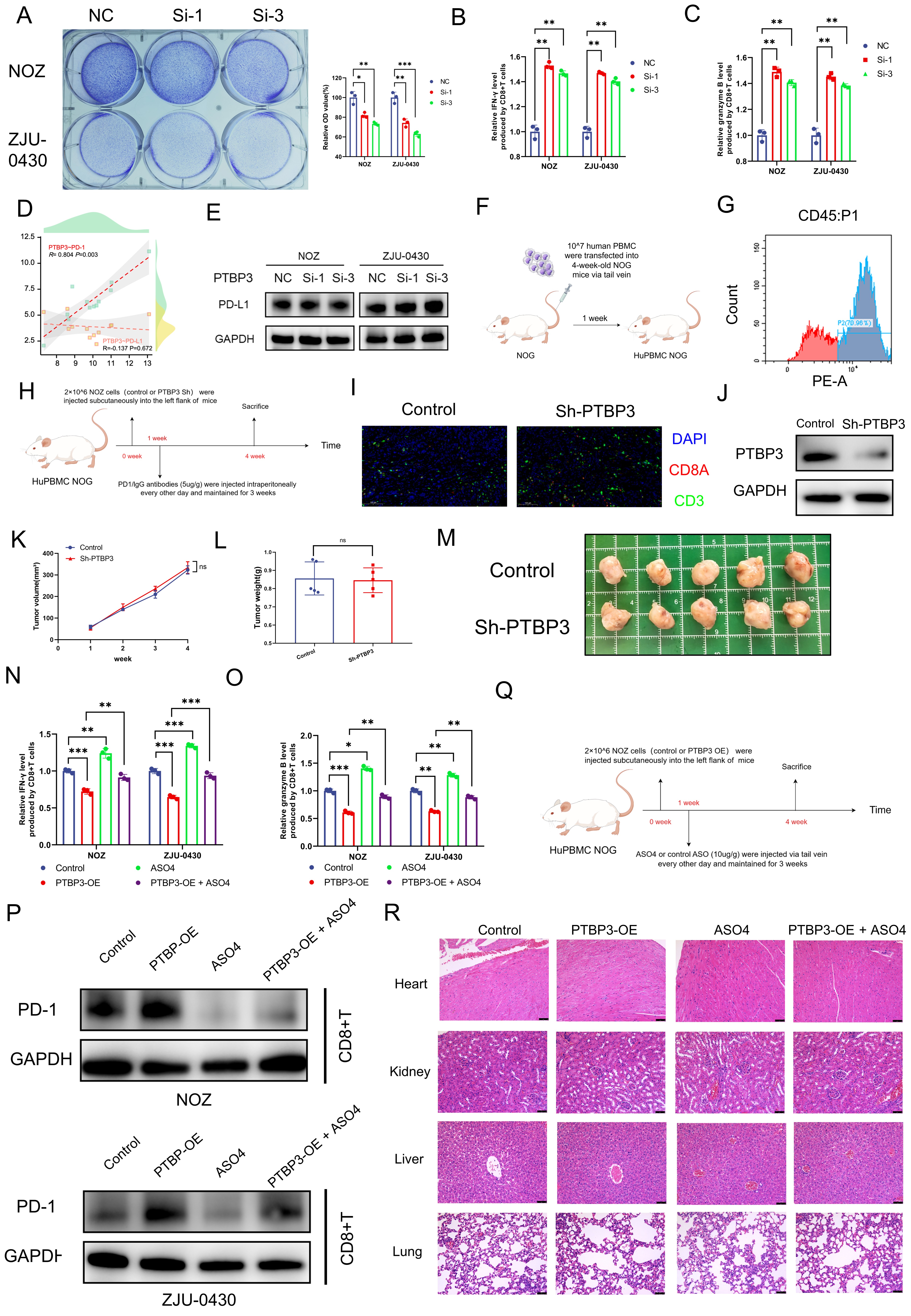


Supplementary Figure3. A. Cells viability as researched by crystal violet staining results show that CD8+T cells killed more tumor cells when co-culturing with PTBP3 konckdown in NOZ and ZJU-0430 cells. In brief, after co-cultivation for 48h removing the debris and T cells by washing with PBS for several times, living cells were quantified using a spectrometer at 570 OD. Then, cells were fixed with 4% paraformaldehyde, and then stained with 0.1% crystal violet. B and C. ELISA assay results show that CD8+T cells produced more IFNγ and granzyme B when co-culturing with PTBP3 konckdown in NOZ and ZJU-0430 cells. D. Analysis of PTBP3 expression correlation with PD-1 and PD-L1 expression based on 12 pairs of GBC mRNA-seq data. E. Effect of PTBP3 knockdown on PD-L1 expression in tumor cells using western blotting. F. Schematic diagram of the method for establishing HuPBMC NOG mice. Briefly, human PBMC cells were injected via tail vein into four-week-old NOG mice to construct the HuPBMC NOG model. G. Flow cytometry was performed to detect the positive rate of human CD45 in the HuPBMC mice model. H. Schematic flow of the in vivo experiment. Briefly, HuPBMC NOG model mice were injected subcutaneously with 2×10^6 NOZ cells (nc or shPTBP3-treated) for one week, followed by intraperitoneal injections of PD-1 antibody or IgG every other day for three weeks. I. Labeling of CD8A and CD3 in subcutaneous tumors using multiplex immunohistochemistry. J. Validation of PTBP3 shRNA knockdown efficiency using western blotting. K. Measurement of subcutaneous tumor growth volume in NOG mice. L. Measurement of tumor weight in NOG mice. M. Illustration of a subcutaneous tumor in NOG mice. N and O. ELISA assay results show that CD8+T cells produced less IFNγ and granzyme B when co-culturing with PTBP3 overexpression in NOZ and ZJU-0430 cells, but the ASO4 treatment saves this reduction. P. Evaluation of PD-1 expression of T cells after PTBP3 overexpression or ASO4 treatment in tumor cells using western blotting (CD8+T cells and tumor cells co-cultured at a ratio of 1:1 for 48 h). Q. Schematic flow of the in vivo experiment. Briefly, HuPBMC NOG model mice were injected subcutaneously with 2×10^6 NOZ cells (control or PTBP3 OE-treated) for one week, followed by tail vein injection of ASO4 or control ASO every other day for three weeks. R. HE staining in the lung, liver, kidney, and heart in four groups. Statistical tests involved: *P<0.05, **P<0.01, Student's t-test; Data are expressed as mean±SD, n=3.

Supplementary figure4


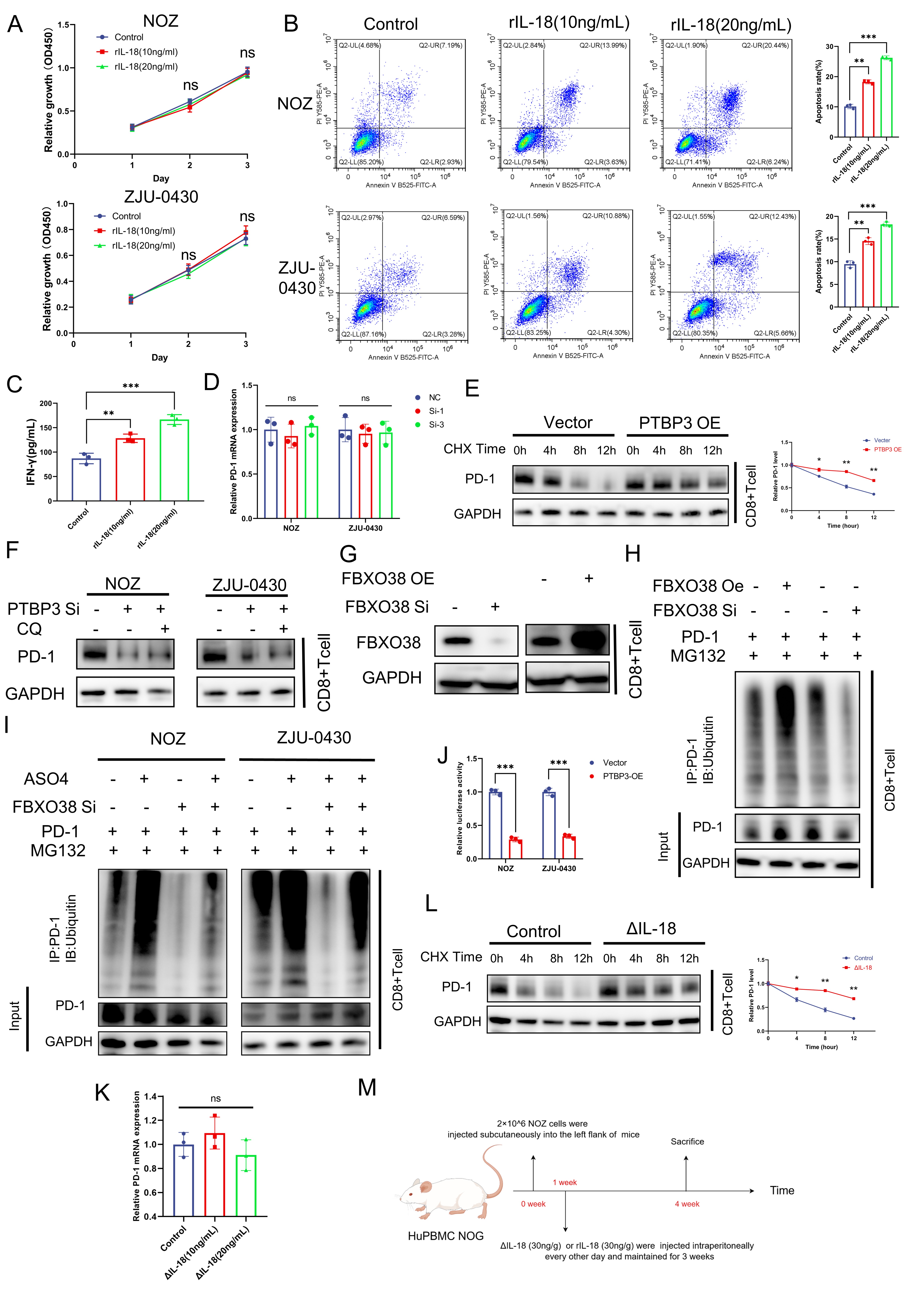


Supplementary Figure4. A. CCK-8 results of the effect of rIL-18 on GBC cell proliferation. “rIL-18” represents human recombinant IL-18. B. Evaluation of tumor cell killing capacity of T cells after rIL-18 treatment in tumor cells using flow cytometry (CD8+T cells and tumor cells co-cultured at a ratio of 1:1 for 48 h). C. ELISA assay to analyze changes in supernatant IFN-γ level after rIL-18 treatment in CD8+T cells. D. Effect of knockdown of PTBP3 in tumor cells on PD-1 mRNA in CD8+T cells. E. CD8+T cells with tumor cell (with or without PTBP3 overexpression) supernatants were treated with 60 μg/ml CHX, then the proteins were extracted according to the time point for detecting PD-1 levels. F. Levels of PD-1 in CD8+T cells with tumor cell (with or without PTBP3 knockdown) supernatants were detected by western blotting after 12 h treatment with CQ (20 μM). G. Validation of FBXO38 knockdown and overexpression efficiency in CD8+T cells using western blotting. H. Exploring the effect of FBXO38 on PD-1 ubiquitination levels using western blotting in CD8+T cells. I. Exploring the effect of ASO4 treatment on PD-1 ubiquitination levels using western blotting in GBC cells. J. Levels of transcriptional activity of FBXO38 in CD8+T cells with or without ΔIL-18 were detected by luciferase reporter assay. K. Effect of ΔIL-18 on PD-1 mRNA in CD8+T cells. L. CD8+T cells with or without ΔIL-18 were treated with 60 μg/ml CHX, then the proteins were extracted according to the time point for detecting PD-1 levels. M. Schematic flow of the in vivo experiment. Briefly, HuPBMC NOG model mice were injected subcutaneously with 2×10^6 NOZ cells for one week, followed by intraperitoneal injections of IL-18 or control ΔIL-18 every other day for three weeks. Statistical tests involved: *P<0.05, **P<0.01, Student's t-test; Data are expressed as mean±SD, n=3.

Supplementary figure5


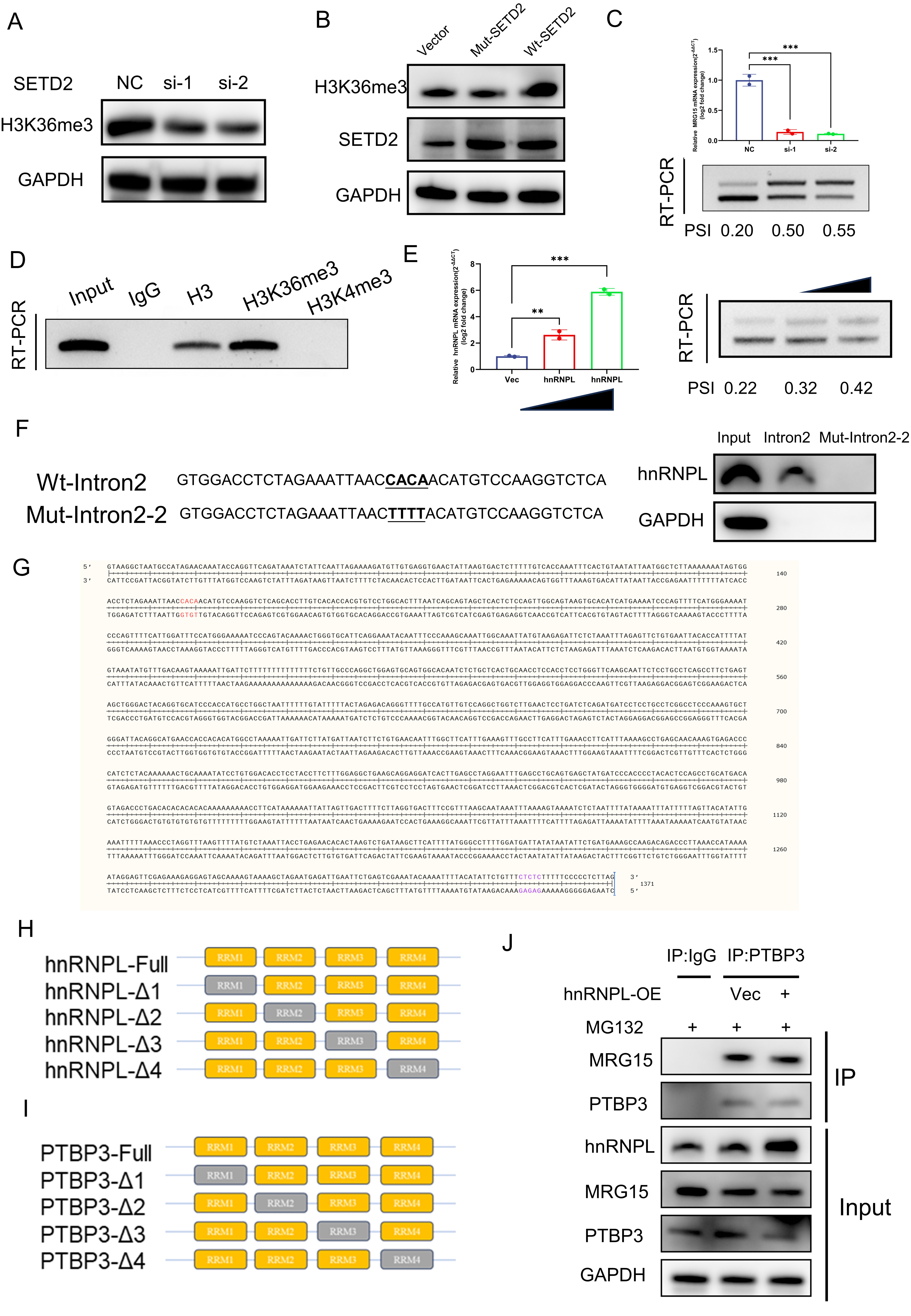


Supplementary Figure5. A. H3K36me3 expression was detected by western blotting after knockdown of SETD2 in NOZ. B. H3K36me3 expression was detected by western blotting after overexpression of Wt-SETD2\Mut-SETD2 in NOZ. C. Validation of the effect of knockdown of MRG15 on exon skipping of IL-18 using RT-PCR assay. D. DNA electrophoresis obtained by CHIP experiments using anti-IgG\H3\H3K36me3\H3K4me3 antibodies. E. RT-PCR experiments after overexpression of hnRNPL. F. The PCR products of PTBP3 binding sites (Wt-Intron2\Mut-Intron2-2) were applied to an in vitro transcription assay with Biotin-labeled. Binding of these RNAs with hnRNPL/GAPDH was detected by an RNA pull-down assay in NOZ cells. G. Location map of hnRNPL binding site in IL-18 pre-mRNA intron2 (red mark) and PTBP3 binding site in IL-18 pre-mRNA intron2 (purple mark). H. Schematic diagram of the different Myc-tagged hnRNPL deletion mutants (gray represents missing domain). I. Schematic diagram of the different Flag-tagged PTBP3 deletion mutants (gray represents missing domain). J. Overexpression of hnRNPL to validate of PTBP3 binding to MRG15 by IP using anti-PTBP3 antibody. Statistical tests involved: *P<0.05, **P<0.01, Student's t-test; Data are expressed as mean±SD, n=3.
